# Progressive multi-scale learning for lightweight YOLO-based multi-disease detection in chest X-ray

**DOI:** 10.3389/fmed.2026.1901057

**Published:** 2026-10-29

**Authors:** Shanweichen Chen, Quanhong Wang, Lihong Zhu, Fengmei Su

**Affiliations:** 1 College of Education, Baoji University of Arts and Sciences, Baoji, China; 2 Radiology Department, 96607 Hospital of the PLA, Baoji, China; 3 School of Computer Science and Technology (School of Artificial Intelligence), Yibin University, Yibin, China; 4 Yibin Engineering Technology Research Center for Intelligent Fluid Measurement and Control, Yibin, China

**Keywords:** chest X-ray, computer-aided diagnosis, explainable AI, multi-disease detection, progressive multi-scale learning

## Abstract

**Background:**

Chest X-ray (CXR) is a primary imaging modality for thoracic disease screening, but manual interpretation of multi-disease cases can be limited by radiologist workload and variability in diagnostic experience, increasing the risk of missed abnormalities, particularly for small lesions. Although deep learning-based detectors have achieved promising performance, many high-accuracy models remain computationally intensive, limiting their deployment in resource-constrained clinical environments. Furthermore, lightweight detectors may face challenges in learning multi-scale lesion representations under fixed-resolution training paradigms.

**Methods:**

We proposed PMSL-YOLO, a lightweight YOLOv8n-based multi-disease detection framework based on Progressive Multi-Scale Learning (PMSL). Different from conventional fixed-resolution training strategies, PMSL introduces a structured progressive learning paradigm that gradually guides the detector from global anatomical representation learning to fine-grained lesion localization through multi-resolution adaptation. The framework incorporates progressive resolution transitions and staged optimization strategies to improve feature representation capability. In addition, CXR-specific augmentation strategies, including optimized Mosaic, MixUp, HSV brightness adjustment, and moderate geometric transformations, were employed to enhance detection of subtle and small lesions.

**Results:**

PMSL-YOLO with the progressive 512→512→640→1024 configuration achieved a Precision of 94.1%, Recall of 92.4%, mAP@0.5 of 93.3%, and mAP@0.5:0.95 of 70.1% on the independent test set, compared with 89.6%, 80.9%, 86.7%, and 68.4% for the 1024 × 1024 baseline YOLOv8n.

**Conclusion:**

PMSL-YOLO provides a lightweight progressive training strategy for multi-disease CXR detection, achieving improved detection performance while preserving the computational characteristics of YOLOv8n. The independent test results provide evidence of the observed performance gains on data not used during model development within the present VinBigData dataset and experimental protocol. However, generalization across institutions, imaging devices, patient populations, and acquisition protocols remains to be established through external validation.

## 1 Introduction

Chest X-ray (CXR) is the most widely used and cost-effective imaging modality for screening and diagnosing thoracic diseases worldwide. Owing to its low radiation exposure, accessibility, and rapid acquisition, CXR plays an indispensable role in detecting various conditions, including pneumonia, pulmonary nodules, cardiomegaly, and pleural effusion (1). With the global aging population and increasing prevalence of chronic cardiopulmonary diseases, accurate identification of multiple coexisting abnormalities within a single chest radiograph has become increasingly important (2). However, in primary healthcare institutions and resource-limited settings, the shortage of experienced radiologists poses substantial challenges to manual interpretation, resulting in reduced diagnostic efficiency, variability in clinical decisions, and an increased risk of missed detection, particularly for subtle and small lesions (3).

Although deep learning techniques have achieved remarkable progress in CXR analysis, several challenges remain unresolved. First, many high-performance models rely on complex architectures with large computational requirements, making them difficult to deploy in edge devices and resource-constrained clinical environments (4). Second, thoracic abnormalities exhibit substantial variations in size, shape, and appearance, while conventional fixed-resolution training strategies may limit the ability of lightweight detectors to learn comprehensive multi-scale lesion representations (5). Third, existing studies have mainly focused on improving detection accuracy, whereas the requirements of training stability, computational efficiency, and practical deployment have received comparatively less attention (6). Therefore, developing a lightweight detection framework capable of progressively learning multi-scale lesion features remains an important research challenge.

The YOLO family has demonstrated considerable potential in medical image analysis due to its high inference speed, lightweight architecture, and favorable accuracy-efficiency tradeoff (7). Among these models, YOLOv8n provides an attractive foundation for resource-efficient medical applications. However, most existing YOLO-based CXR studies rely on fixed-resolution training or conventional multi-scale augmentation, which may not fully exploit the hierarchical characteristics of thoracic abnormalities. In particular, small lesions require both global anatomical understanding and fine-grained spatial feature extraction, suggesting the need for a more structured learning process rather than isolated resolution changes.

To address these challenges, we propose PMSL-YOLO, a lightweight multi-disease detection framework based on Progressive Multi-Scale Learning (PMSL). Unlike conventional multi-scale training strategies that independently introduce different image resolutions, PMSL establishes a structured coarse-to-fine learning pathway, enabling the model to gradually transition from global anatomical representation learning to detailed lesion localization. By progressively increasing input resolution and optimizing model adaptation during training, PMSL improves the capability of lightweight detectors to capture abnormalities across different spatial scales while maintaining computational efficiency.

Specifically, PMSL adopts a progressive resolution learning strategy from 512 × 512 to 640 × 640 and finally to 1024 × 1024, together with staged optimization and CXR-oriented augmentation strategies. This design allows the model to first acquire stable global representations and subsequently refine fine-grained lesion features. The proposed framework aims to provide a practical solution for accurate and efficient multi-disease CXR analysis in resource-limited clinical environments.

The main contributions of this study are summarized as follows:

(1) A lightweight Progressive Multi-Scale Learning (PMSL) framework is proposed for CXR multi-disease detection, which introduces a structured coarse-to-fine learning paradigm to improve multi-scale lesion representation.
(2) A progressive training strategy combining resolution adaptation and staged optimization is developed to enhance detection performance and training stability without increasing inference complexity.
(3) Extensive experiments on 14 thoracic abnormality categories within the VinBigData dataset and the present evaluation protocol demonstrate that PMSL-YOLO achieves improved detection performance compared with the evaluated baseline and representative lightweight YOLO-family models while maintaining deployment efficiency. Furthermore, an explainability component based on Grad-CAM is integrated to provide qualitative *post-hoc* visualization of model predictions and support model behavior analysis.

## 2 Related work

### 2.1 Deep learning for CXR detection

CXR, as the most commonly used and cost-effective imaging modality in clinical practice, plays a critical role in the screening, diagnosis, and follow-up of various thoracic diseases, including pneumonia, pulmonary nodules, cardiomegaly, and pleural effusion. With the rapid advancement of deep learning technologies, convolutional neural network (CNN) - based models have achieved substantial progress in CXR analysis. Early studies primarily employed classical architectures such as Visual Geometry Group (VGG), Residual Network (ResNet), Densely Connected Convolutional Network (DenseNet), and EfficientNet, focusing mainly on image-level classification tasks for single or multi-label disease diagnosis, including pneumonia, COVID-19, and pulmonary tuberculosis (8).

In recent years, research emphasis has gradually shifted toward multi-disease joint detection and lesion localization. Large-scale public datasets such as Chestx-ray14, CheXpert, MIMIC-CXR, and VinBigData have provided critical support for this transition.

Recent reviews have summarized the rapid development of deep learning-based chest disease detection, highlighting the progress of CNN-based models, publicly available datasets, preprocessing strategies, and remaining challenges in clinical translation (9).

Beyond image-level classification, researchers have increasingly explored object detection approaches to provide explicit lesion localization. Two-stage detectors, such as Faster R-CNN and Mask R-CNN (10, 11), first generate candidate regions and then perform region-level classification and refinement, providing strong localization accuracy but generally requiring greater computational cost. More recently, transformer-based detectors, including Detection Transformer (DETR) and its subsequent variants (12), have introduced global attention mechanisms and set-based prediction to model long-range dependencies without relying exclusively on conventional convolutional detection pipelines. Researchers have also explored transformer-based object detection for localized disease detection in chest X-rays, demonstrating the potential of global contextual representation for CXR analysis (13). These approaches have demonstrated the potential of combining accurate lesion localization with richer contextual representation, but their computational complexity and training requirements can remain challenging for resource-constrained medical imaging applications. Consequently, the selection of an appropriate detection framework requires balancing localization accuracy, computational efficiency, and the ability to capture lesions with substantially different spatial scales.

These datasets enable simultaneous annotation and modeling of multiple thoracic abnormalities, including cardiomegaly, atelectasis, pulmonary nodules, and pneumonia, thereby significantly advancing multi-label detection methodologies. Under laboratory conditions, existing approaches have achieved impressive performance, with many models demonstrating Area Under the Curve (AUC) values comparable to or even surpassing radiologist-level performance (14). Furthermore, transfer learning, attention mechanisms, and ensemble learning strategies have been incorporated to improve recognition of small lesions and complex overlapping abnormalities (15).

Despite these advancements, current deep learning methods still face substantial challenges in real-world deployment. First, most high-performance models are computationally intensive and parameter-heavy, limiting their applicability in primary healthcare settings and resource-constrained environments. Second, insufficient model interpretability restricts the ability to provide intuitive lesion localization evidence, thereby reducing clinical trust. Third, generalization across imaging devices and diverse patient populations remains inadequate. More importantly, the inherent characteristics of CXR images, including low contrast, overlapping anatomical structures, and large variation in lesion scales, make accurate localization of small abnormalities particularly challenging for lightweight detection models. These limitations hinder the effective translation of laboratory achievements into widespread clinical practice.

### 2.2 Lightweight YOLO in medical imaging

The YOLO family of object detection algorithms has gained extensive attention in medical imaging analysis due to its single-stage architecture, end-to-end training paradigm, and real-time inference capability (16). Compared with many two-stage and transformer-based detectors, YOLO models provide a relatively efficient single-stage detection framework, offering a favorable balance between localization accuracy, computational efficiency, and inference speed for medical imaging applications. This characteristic makes YOLO-based models particularly attractive for clinical decision-support applications and resource-constrained deployment scenarios.

In the domain of CXR analysis, YOLO has been widely applied to the detection of pneumonia, pulmonary tuberculosis, COVID-19, pulmonary nodules, and multiple thoracic abnormalities. Studies based on datasets such as VinBigData have demonstrated that YOLO-based models can effectively perform simultaneous localization and classification of multi-category lesions, achieving strong performance in metrics such as mean Average Precision (mAP), Precision, and Recall (17). By improving feature fusion mechanisms, incorporating attention modules, or optimizing loss functions, several studies have further enhanced the detection capability for small lesions and low-contrast regions (18).

Nevertheless, most existing research primarily focuses on medium-to-large variants such as YOLOv8 m and YOLOv8 L (19), which require substantial computational resources and larger parameter capacities, thereby constraining deployment in mobile devices, edge computing systems, and primary healthcare environments.

Recent lightweight detectors, including YOLOv10 and YOLO11 variants, have further improved the efficiency–accuracy trade-off through architectural optimization and lightweight design strategies (20) (21),; however, their performance still depends on conventional fixed-resolution training paradigms.

Although lightweight YOLO models, such as YOLOv8n, offer significant advantages in deployment flexibility, their detection performance, particularly sensitivity to small targets, remains suboptimal when addressing the pronounced scale heterogeneity of lesions in chest radiographs (22). Current lightweight YOLO applications in medical imaging predominantly rely on fixed-resolution training strategies and lack systematic optimization approaches specifically designed for multi-scale lesion detection (23). Therefore, improving the multi-scale representation capability of lightweight detectors through training strategy optimization, rather than increasing model complexity, represents an important research direction.

### 2.3 Progressive and multi-scale learning

Lesions in CXR images exhibit substantial scale variability, ranging from large-scale abnormalities such as cardiomegaly and pleural effusion to small lesions, including pulmonary nodules and calcifications. Traditional fixed-resolution training strategies often struggle to simultaneously balance global anatomical structure modeling and fine-grained lesion recognition (24).

Multi-scale learning enhances model adaptability to targets of varying sizes through different input resolutions, multi-level feature representations, or feature fusion mechanisms, such as Feature Pyramid Network (FPN) and Path Aggregation Network (PAN). Such multi-scale representation strategies have been explored in both convolutional and hybrid detection frameworks to capture contextual information at different spatial granularities. This approach has demonstrated strong effectiveness in both natural image detection and medical imaging tasks, including pulmonary nodule detection and breast lesion analysis (25).

However, most existing multi-scale approaches mainly focus on architectural feature fusion or simultaneous scale representation, while providing limited consideration of how the training process itself should progressively adapt to lesion-scale variation.

Progressive learning further emphasizes an ordered coarse-to-fine training paradigm, in which the model initially learns global structural information at lower resolutions and subsequently transitions to higher resolutions to strengthen detailed feature representation. This strategy can reduce the complexity of early-stage optimization, improve training stability, and enable more effective focus on fine-grained features at high resolution (26).

In the context of multi-disease detection from CXR, PMSL offers distinct advantages. By organizing training from coarse to fine, the model can rapidly learn global thoracic anatomical structures and large-scale lesions during the low-resolution stage, enhance representation of medium-scale abnormalities during intermediate-resolution stages, and ultimately improve precise detection of small lesions and complex boundaries during high-resolution stages.

Integrating a staged Freeze–Unfreeze backbone training mechanism can further improve feature transfer efficiency and stabilize the training process. Different from conventional multi-scale feature fusion methods or random-resolution training strategies, PMSL introduces a task-oriented progressive optimization procedure that coordinates resolution transition and backbone adaptation specifically for lightweight CXR detection. Although progressive multi-scale strategies have been explored in general object detection tasks, systematic research on their application within lightweight YOLO frameworks for multi-disease CXR detection remains relatively limited. In particular, few studies have investigated how progressive resolution learning can improve small-lesion sensitivity while maintaining the computational advantages of lightweight detectors.

## 3 Materials and data preparation

### 3.1 Dataset description

This study utilizes the VinBigData CXR Abnormalities Detection dataset as the experimental data source (27). It is one of the largest and highest-quality publicly available datasets in the field of multi-disease CXR analysis. The dataset was jointly collected by multiple major medical institutions in Vietnam and meticulously annotated by professional radiologists.

The original release contains approximately 18,000 posterior-anterior (PA) and anterior-posterior (AP) chest radiographs stored in DICOM format with varying resolutions. After data quality screening, annotation preprocessing, and removal of unsuitable samples (including images with incomplete annotations or invalid bounding boxes), 15,000 images were retained as the final dataset. These 15,000 images were subsequently partitioned into 12,000 training images, 1,500 validation images, and 1,500 untouched test images to enable independent evaluation while preserving a sufficient training sample size.

This study focuses on the detection of 14 categories of thoracic abnormalities. Each image may contain multiple lesion regions, making the task a typical multi-label and multi-object detection problem. During preprocessing, images containing only the “No finding” label were excluded, as the present work concentrates on multi-disease abnormality detection rather than normal/abnormal classification. Cases originally labeled as “Other” were retained and mapped to the final “Other lesion” category according to the processed YOLO-compatible annotation scheme.

The final 14 abnormality categories used for model training and evaluation are: aortic enlargement, atelectasis, calcification, cardiomegaly, consolidation, interstitial lung disease (ILD), infiltration, lung opacity, nodule, other lesion, pleural effusion, pleural thickening, pneumothorax, and pulmonary fibrosis. The original VinBigData annotation contains 14 radiologist-defined abnormality categories. During preprocessing, category names were reorganized according to the official annotation mapping and YOLO-compatible label requirements. The final label set used for model training is summarized in Table 4.

### 3.2 Annotation processing

The original VinBigData dataset provides lesion annotations in CSV format, whereas YOLO-based object detection models require a specific annotation format. Therefore, this study designs a complete annotation conversion pipeline to ensure high-quality adaptation of the data to the YOLOv8 training framework.

The annotation preprocessing pipeline consists of three main procedures: image normalization, coordinate transformation, and YOLO-format label generation. These procedures ensure that the original radiologist-provided annotations are accurately converted into a format compatible with object detection training while preserving spatial information of lesion regions.

#### 3.2.1 Image normalization and coordinate transformation

The original CXR images exhibit significant variability in resolution, which is not suitable for batch training. In this study, all images are first resized to a predefined target resolution. The corresponding bounding box coordinates are then linearly rescaled to maintain spatial consistency, as shown in Equations 1 and 2:

$$x^{\prime }=x\times \frac{W_{\mathrm{target}}}{W_{\mathrm{original}}}$$ (1)

$$y^{\prime }=y\times \frac{H_{\mathrm{target}}}{H_{\mathrm{original}}}$$ (2)

Where $W_{\mathrm{target}}$ and $H_{\mathrm{target}}$ denote the target image dimensions, while $W_{\mathrm{original}}$ and $H_{\mathrm{original}}$ represent the original image dimensions. All transformed coordinates are further clipped to ensure they remain within valid image boundaries.

#### 3.2.2 YOLO format label generation

The converted annotations are stored in the standard YOLO format, where each line corresponds to a single object:

$$\mathrm{class}\_\mathrm{id},\;x_{\mathrm{center}},\;y_{\mathrm{center}},\;\mathrm{width},\;\mathrm{height}$$

Where, $x_{\mathrm{center}}\;$ and $y_{\mathrm{center}}$ denote the normalized center coordinates of the bounding box, while width and height represent the normalized width and height. All values are normalized to the range [0,1]. For each image, an independent.txt label file is generated and stored according to the predefined training and validation splits.

The converted annotations preserve the original lesion localization information provided by radiologists, enabling consistent comparison among baseline models, ablation experiments, and the proposed PMSL-YOLO framework.

### 3.3 Duplicate label removal

The VinBigData dataset was annotated independently by multiple radiologists. As a result, the same lesion often has multiple highly overlapping yet slightly inconsistent bounding boxes. While such multi-expert annotations reflect clinical consensus, they may introduce noise that adversely affects training stability and convergence behavior.

To address this issue, a duplicate annotation refinement strategy based on class-wise grouping, IoU-based clustering, and bounding box fusion is applied as a deterministic dataset-level preprocessing step before the final train–validation–test partition. The purpose of this procedure is to normalize highly overlapping annotations referring to the same lesion rather than to remove clinically distinct lesions. Fusion is therefore performed only among annotations belonging to the same abnormality category and satisfying a predefined spatial-overlap criterion.

#### 3.3.1 Class-wise grouping

All bounding boxes within the same image are first grouped according to their category labels. Duplicate removal is performed only within each class-specific group to avoid mistakenly merging distinct lesions. This restriction is necessary because bounding boxes from different disease categories may overlap spatially while representing different pathological findings.

#### 3.3.2 IoU-based clustering

For each group, the Intersection over Union (IoU) between any two bounding boxes is computed using Equation 3:

$$IoU=\frac{\mathrm{Area}(B_{1}\cap B_{2})}{\mathrm{Area}(B_{1}\cup B_{2})}$$ (3)

IoU is used as the clustering criterion because it provides a direct and interpretable measure of spatial overlap between two bounding boxes. When two annotations of the same category exhibit substantial spatial overlap, they are considered likely to correspond to the same underlying lesion. In the implemented preprocessing pipeline, the IoU threshold is set to 0.6. Specifically, two same-class bounding boxes with an IoU greater than or equal to 0.6 are assigned to the same duplicate cluster.

The threshold of 0.6 was selected as the default configuration because it provides a relatively strict overlap criterion: annotations must share a substantial portion of their spatial extent before being considered duplicates. This setting is intended to reduce redundant expert annotations while avoiding excessive merging of spatially adjacent but potentially distinct lesions.

#### 3.3.3 Fused bounding box generation

For each cluster of duplicated boxes, a final representative bounding box is generated using coordinate-wise averaging, as shown in Equations 4–7:

$$x_{\mathrm{fused}}=\mathrm{mean}(x_{i})$$ (4)

$$y_{\mathrm{fused}}=\mathrm{mean}(y_{i})$$ (5)

$$w_{\mathrm{fused}}=\mathrm{mean}(w_{i})$$ (6)

$$h_{\mathrm{fused}}=\mathrm{mean}(h_{i})$$ (7)

Coordinate-wise averaging is adopted to obtain a representative spatial estimate from multiple annotations referring to the same lesion. Rather than arbitrarily retaining one annotator's bounding box, averaging the corresponding coordinates provides a simple and reproducible representation of the common spatial extent of the overlapping annotations. Because fusion is restricted to same-class boxes satisfying the IoU criterion, this procedure is designed to reduce minor inter-annotator localization discrepancies while preserving the identity and approximate extent of the lesion. This fusion strategy preserves multi-expert consensus information while effectively reducing annotation variance and improving label consistency.

The resulting fused annotations are subsequently used consistently for all baseline, ablation, and PMSL experiments. The fusion procedure is applied as a deterministic dataset-level annotation preprocessing step before dataset partitioning and does not use model predictions, validation performance, or test-set performance information. The same preprocessing rule is applied consistently to the training, validation, and untouched test subsets. The untouched test subset is not used for IoU-threshold selection, model training, checkpoint selection, hyperparameter tuning, or any other model-development decision. Therefore, it does not constitute model-specific post-processing or performance-oriented label adjustment.

#### 3.3.4 Sensitivity analysis of the IoU threshold

To examine the sensitivity of the annotation fusion procedure, three representative IoU thresholds (0.4, 0.6, and 0.8) were evaluated under the same training and validation protocol, model architecture, and random seed as the main experiments. The results are summarized in Table 1.

**Table 1:** Sensitivity analysis of the ioU threshold for annotation fusion.

IoU Threshold	Training Boxes After Fusion	Precision (%)	Recall (%)	mAP@0.5 (%)	mAP@0.5:0.95 (%)
0.4	27,760	92.3	86.4	90.6	65.6
0.6	28,892	94.5	89.2	91.4	70.3
0.8	29,480	94	85.7	89.9	64.8

All metrics were obtained from independent training runs under the progressive PMSL configuration. Minor differences from the main results in Table 7 are expected due to independent optimization trajectories.

As shown in Table 1, the 0.6 threshold achieves the highest Precision, Recall, mAP@0.5, and mAP@0.5:0.95 while retaining 28,892 training boxes. Therefore, 0.6 was adopted as the default IoU threshold for all subsequent experiments. Note that the absolute metric values differ slightly from the main experimental results because each threshold setting was evaluated in an independent training run; the relative ranking among thresholds remains consistent. A more exhaustive search could be explored in future work.

### 3.4 Data augmentation strategy

CXR images exhibit complex lesion morphology and significant scale variability. In particular, small lesions such as pulmonary nodules, micro-calcifications, and localized infiltrations are often characterized by limited spatial extent and indistinct boundaries, making them highly susceptible to class imbalance and variations in image quality. To improve model generalization and robustness, this study designs a data augmentation strategy tailored to chest radiography characteristics under the proposed PMSL framework.

Unlike conventional natural-image augmentation strategies that primarily aim to increase visual diversity, CXR augmentation must preserve anatomical plausibility and pathological consistency. Therefore, a combination of spatial-domain and intensity-domain transformations is applied while avoiding operations that may introduce unrealistic anatomical deformation. All data augmentation operations described in this section are applied exclusively to the training set. The validation and untouched test sets remain completely free of training-time augmentation and are evaluated using their original image content and annotations, thereby avoiding augmentation-induced distribution shifts and potential data leakage during model evaluation.

The employed augmentation techniques include Mosaic augmentation, MixUp augmentation, HSV brightness adjustment, and moderate geometric transformations. These augmentation operations are consistently applied to the training portion of all experimental settings, including baseline models, ablation experiments, and PMSL training, while the validation and test sets remain unaugmented to ensure a consistent and fair evaluation protocol.

#### 3.4.1 Mosaic augmentation

Mosaic augmentation constructs a new training sample by randomly combining four images into a single composite image, thereby introducing diverse lesion distributions and enabling the model to learn from multi-scale objects and complex backgrounds (28).

In this study, an adaptive Mosaic probability is adopted across different training stages. Specifically, during Stage 1 (512 × 512 resolution), the Mosaic probability is set to 0.90; during Stage 2 (640 × 640 and 1024 × 1024 resolutions), it is increased to 0.95.

The primary advantage of this strategy lies in increasing the diversity of lesion-scale distributions within training samples, thereby improving the model's capability to detect coexisting multi-lesion scenarios. It enhances spatial diversity and effectively alleviates class imbalance. Notably, the higher Mosaic probability at higher resolutions further strengthens model robustness in handling complex thoracic structures.

#### 3.4.2 Mixup augmentation

MixUp augmentation linearly interpolates between two images and their corresponding labels, encouraging the model to learn smoother decision boundaries. In CXR analysis, this approach helps reduce overfitting and improves the stability of model predictions for low-contrast lesions.

In this study, an adaptive MixUp probability is also employed. The probability is set to 0.08 at the 512 × 512 stage and increased to 0.12 at the 640 × 640 and 1024 × 1024 stages.

A relatively low MixUp ratio helps prevent excessive blurring of lesion regions while still introducing moderate sample complexity. This strategy contributes to mitigating overfitting, improving tolerance to ambiguous lesion boundaries, and enhancing cross-patient generalization.

#### 3.4.3 HSV brightness augmentation

To simulate variations in imaging devices, exposure conditions, and acquisition protocols, brightness perturbation is applied in the HSV color space by modifying only the V channel, while keeping the H and S channels unchanged.

The parameter settings are defined as follows: hsv_v is set to 0.35 in Stage 1 and increased to 0.42 in Stage 2, while hsv_h and hsv_s are both set to 0.0.

Given that chest radiographs are inherently grayscale images, this brightness-only augmentation avoids introducing clinically implausible color distortions while improving robustness to variations in imaging conditions across different devices and acquisition settings.

#### 3.4.4 Geometric augmentation strategy

To simulate variations in patient posture, imaging angles, and acquisition devices, this study employs moderate geometric transformations, including translation (range: 0.10–0.15), scaling (range: 0.45–0.50), and horizontal flipping (fliplr probability: 0.5).

Translation augmentation improves the model's robustness to spatial shifts in lesion location, while scaling augmentation enhances its ability to adapt to lesions of varying sizes. Horizontal flipping further increases sample diversity by incorporating variations in left–right thoracic anatomy.

To preserve clinical realism, geometric operations that may distort anatomical structures, such as rotation and shearing, are strictly constrained and not applied. This conservative augmentation design aims to improve robustness without compromising the anatomical characteristics required for reliable CXR lesion localization.

#### 3.4.5 Stage-wise augmentation design

To better align with the proposed PMSL strategy, augmentation parameters are dynamically adjusted across different training stages, as summarized in Table 2.

**Table 2:** Stage-wise data augmentation settings.

Stage	Resolution	Mosaic	MixUp	HSV-V	Scale
Stage 1	512 × 512	0.9	0.08	0.35	0.45
Stage 2-1	512 × 512	0.9	0.08	0.35	0.5
Stage 2-2	640 × 640	0.95	0.12	0.42	0.5
Stage 2-3	1024 × 1024	0.95	0.12	0.42	0.5

This stage-wise design enables the model to stably learn global anatomical structures in the early stage, progressively enhance scale adaptability in intermediate stages, and strengthen fine-grained detection of small lesions and complex boundaries in the final high-resolution stage. Throughout all experiments, these augmentation operations are restricted exclusively to the training set; validation and test images are evaluated without any Mosaic, MixUp, HSV perturbation, or geometric augmentation. The augmentation configuration remains identical across all comparative experiments, ensuring that observed performance differences primarily reflect the contribution of the proposed training strategy rather than inconsistent preprocessing conditions.

### 3.5 Dataset partition and experimental protocol

To ensure scientific rigor, reproducibility, and fair comparison among different experiments, this study adopts a strictly independent image-level partitioning strategy to divide the retained VinBigData dataset into training, validation, and untouched test subsets.

The dataset split follows four principles:

(1) complete independence between training, validation, and test sets to prevent data leakage;
(2) image-level partitioning, ensuring that the same image does not appear in more than one subset;
(3) an untouched test set that is excluded from training, validation-based model selection, hyperparameter tuning, checkpoint selection, and stage-to-stage initialization throughout the entire experimental process;
(4) a unified fixed split scheme applied consistently across all experiments, including baseline models, ablation studies, SOTA comparisons, and the full PMSL strategy.

The original VinBigData dataset contains approximately 18,000 chest radiographs. After removing images with incomplete annotations, invalid bounding boxes, or unsuitable samples during annotation preprocessing, 15,000 images were retained for the final experiments.

The retained 15,000 images were partitioned into 12,000 training images, 1,500 validation images, and 1,500 untouched test images using a fixed-seed stratified sampling strategy. The training set was used for model optimization, the validation set was used exclusively for model monitoring and development-stage model selection, and the 1,500-image untouched test set was completely isolated from the model-development process. It was accessed only for the final evaluation after all training configurations and model-selection decisions had been finalized. The detailed statistics are presented in Table 3.

**Table 3:** Dataset split statistics.

Subset	Image Count	Lesion Images	Bounding Boxes
Training Set	12,000	3518	28,892
Validation Set	1,500	442	3598
Untouched Test Set	1,500	434	3606
Total	15,000	4394	36,096

The VinBigData dataset exhibits notable class imbalance. After partitioning, we conducted a strict distribution consistency check across all disease categories in the training, validation, and untouched test sets to characterize the class distribution and ensure that no substantial category imbalance was introduced by the partitioning procedure. The detailed distribution is shown in Table 4.

**Table 4:** Class distribution across training, validation, and untouched test sets.

ID	Class Name	Train Count	Val Count	Test Count	Consistency
0	Aortic enlargement	5,729	717	716	✓
1	Atelectasis	223	26	30	✓
2	Calcification	768	94	98	✓
3	Cardiomegaly	4,342	543	542	✓
4	Consolidation	445	56	55	✓
5	ILD	800	98	102	✓
6	Infiltration	998	125	124	✓
7	Lung Opacity	2,001	244	238	✓
8	Nodule	2,064	256	260	✓
9	Other lesion	1,762	221	220	✓
10	Pleural effusion	1,981	248	247	✓
11	Pleural thickening	3,874	481	487	✓
12	Pneumothorax	181	23	22	✓
13	Pulmonary fibrosis	3,724	466	465	✓

To further improve experimental reproducibility, the final partition indices were fixed and saved as train_ids.pkl, val_ids.pkl, and test_ids.pkl. All experiments were conducted using the same dataset split, ensuring that performance differences among baseline models, ablation variants, and PMSL-YOLO were caused by methodological changes rather than random partition variations. The test indices were fixed before model training and were not accessed for training, validation-based model selection, hyperparameter tuning, or checkpoint selection.

During training, the validation set was evaluated after each epoch to monitor model performance using Precision, Recall, mAP@0.5, and mAP@0.5:0.95. The validation set was not used for gradient-based parameter updates; however, its performance was repeatedly considered during checkpoint selection throughout the progressive training process. Specifically, the best-performing checkpoint at each stage was selected and used to initialize the subsequent stage. This validation-based model-selection procedure was applied exclusively to the training and validation sets and did not involve the untouched test set.

The untouched test set was strictly excluded from all model-development procedures. Specifically, test images and their annotations were not used for gradient-based optimization, hyperparameter tuning, augmentation-parameter selection, checkpoint selection, early-stopping decisions, or stage-to-stage initialization. The test set was accessed only after the training configuration and model-selection procedures had been finalized, and its results were used for the final evaluation of the selected models. Therefore, the test-set performance provides an evaluation of model behavior on images that remained completely unseen throughout the model-development process.

The same fixed validation and untouched test sets were retained across all baseline, ablation, and PMSL experiments to ensure a consistent basis for comparison. The validation set supported model development and comparative analysis, whereas the untouched test set was reserved exclusively for final evaluation after all model-development decisions had been completed.

## 4 Proposed methodology

### 4.1 Baseline YOLOv8n framework

This study adopts Ultralytics YOLOv8n as the baseline model. YOLOv8n is the most lightweight variant within the YOLOv8 family. Compared with medium- and large-scale versions such as YOLOv8 m and YOLOv8 L, YOLOv8n offers significant advantages in terms of parameter count and floating-point operations (FLOPs), substantially reducing inference overhead while maintaining competitive detection accuracy. These characteristics provide a strong foundation for practical clinical deployment, particularly in primary healthcare environments where computational resources are limited. Its compact architecture and reduced parameter size provide a suitable foundation for efficient inference and deployment in multi-disease CXR detection tasks under resource-constrained settings. Actual inference latency, throughput, and memory consumption are further evaluated experimentally in Section 5.2.

To comprehensively evaluate the impact of input resolution on model performance, two baseline configurations are established:

- Baseline512: Input resolution of 512 × 512, designed to evaluate performance under lightweight and computationally efficient conditions;
- Baseline1024: Input resolution of 1024 × 1024, used to assess the upper-bound performance under high-resolution input settings.

Both baseline models are initialized with the official pretrained weights and fine-tuned on the VinBigData dataset for 14-category thoracic abnormality detection. YOLOv8n employs a single-stage anchor-free detection paradigm, enabling simultaneous object localization and classification while effectively minimizing computational complexity.

The YOLOv8n architecture mainly consists of three components: Backbone, Neck, and Detection Head. The Backbone is responsible for hierarchical feature extraction; the Neck utilizes a PAN structure for multi-scale feature fusion; and the Detection Head adopts a Decoupled Head design to separately process classification and regression tasks, while incorporating Distribution Focal Loss (DFL) to improve localization precision. The detailed module composition and parameter settings are summarized in Table 5.

**Table 5:** Main modules and parameter settings of the YOLOv8n baseline model.

Module Type	Main Structure/Parameters	Output Feature Map Size	Parameters
Input Layer	Input image size	512 × 512 × 3	0
Backbone Stage 1	Conv+C2f (×1)	256 × 256 × 64	0.1M
Backbone Stage 2	Conv+C2f (×2)	128 × 128 × 128	0.4M
Backbone Stage 3	Conv+C2f (×4) + SPPF	64 × 64 × 256	1.1M
Backbone Stage 4	Conv+C2f (×4)	32 × 32 × 512	0.5M
Neck (Feature Fusion)	Upsample+C2f + Concat+PAN	Multi-scale fusion (P3/P4/P5)	0.8M
Detection Head	Decoupled Head (classification+regression+DFL)	3 detection layers (80 × 80, 40 × 40, 20 × 20)	0.3M
Total	Complete YOLOv8n model	–	3.2M

YOLOv8n adopts an anchor-free mechanism and Decoupled Head design. The C2f module serves as the primary feature extraction unit, while SPPF is used for multi-scale feature aggregation. Parameter counts are approximate.

Figure 1 presents the overall framework of the proposed PMSL-YOLO system for multi-disease CXR detection. The framework consists of two main phases: offline progressive training and online inference deployment. During training, the YOLOv8n detector is optimized through the proposed Progressive Multi-Scale Learning strategy. During inference, input CXR images are processed through preprocessing, model prediction using the PMSL-trained detector, post-processing, and visualization modules to generate auxiliary diagnostic outputs.

**Figure 1:**
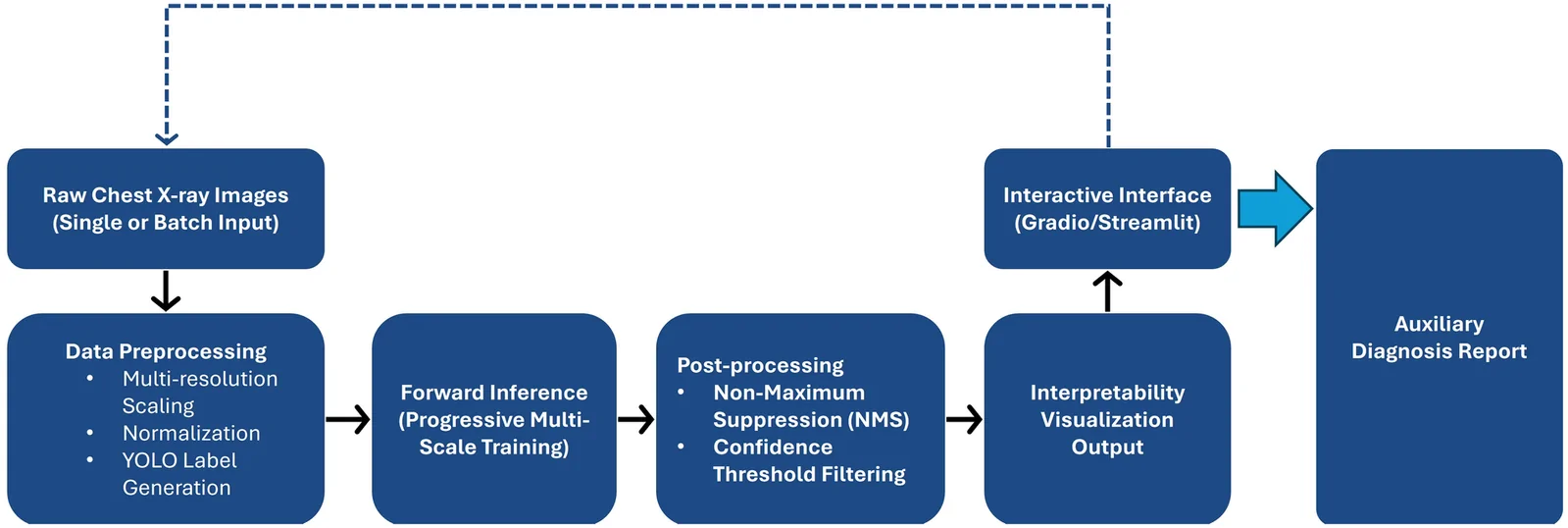
Overall framework of the proposed PMSL-YOLO system for multi-disease CXR detection.

### 4.2 Lightweight optimization strategy

To enhance deployment efficiency and practical usability while preserving detection performance, this study systematically designs a lightweight optimization strategy based on the YOLOv8n baseline model for low-computation environments. Although model training is conducted on high-performance hardware, the framework is developed with the core objectives of lightweight design, ease of deployment, and transferability, making it particularly suitable for primary healthcare institutions and edge computing scenarios.

From the perspective of training optimization, an in-memory caching strategy is adopted to reduce disk I/O overhead, while data loading worker threads are carefully configured to maximize hardware parallelism. In addition, a cosine annealing learning rate scheduler is introduced to ensure smooth learning rate decay, thereby improving convergence stability and training efficiency.

Considering the characteristics of CXR imaging, such as grayscale single-channel input, a high proportion of small lesions, and sensitivity to subtle texture details, this study further refines the default YOLOv8 data augmentation parameters. By restricting excessive color perturbations and aggressive geometric transformations that may compromise medical semantics, while strategically integrating advanced augmentation techniques such as Mosaic and MixUp, the framework effectively improves generalization capability and small-lesion detection performance within a lightweight architecture while preserving clinical realism.

Importantly, the proposed framework maintains lightweight parameter efficiency while supporting cross-platform deployment. The trained model can achieve real-time inference not only on high-performance GPUs but also on standard GPUs, CPUs, and even edge devices. This design philosophy emphasizes that “lightweight” is not solely defined by extreme computational minimization, but rather by achieving consistently efficient performance and reduced resource consumption across diverse hardware environments.

Algorithm 1 **Progressive Multi-Scale Learning Procedure of PMSL-YOLO for CXR Multi-Disease Detection**

**Algorithm**

***Input:***

 • Training dataset *D=(I,Y)*

 • Pretrained YOLOv8n model *M*

**Algorithm**

***Input:***

 • Training dataset *D=(I,Y)*

 • Pretrained YOLOv8n model *M*

 • Progressive resolution stages *R*=[512,512,640,1024]

***Output:***

 • Optimized PMSL-YOLO model *M*

 01: Initialize YOLOv8n model M with pretrained weights

 02: Define progressive training stages:

  Stage 1: 512×512

  Stage 2: 512×512

  Stage 3: 640×640

  Stage 4: 1024×1024

 03: For each training stage i in {1,2,3,4} do:

 04: Resize training images according to current resolution *R_i_*

 05: If i=1 then

 06:  Freeze first 10 backbone layers

 07: Else

 08:  Unfreeze the entire network

 09: End if

 10: Train model using current resolution and CXR-specific augmentation

 11: Save the best checkpoint of current stage

 12: Initialize the next stage using the previous best checkpoint

 13: End for

 14: Return the final optimized PMSL-YOLO model *M*

### 4.3 PMSL strategy

To effectively improve detection capability across lesions of varying scales, particularly for small targets such as pulmonary nodules and subtle infiltrations, this study proposes a PMSL strategy. Different from conventional multi-scale training strategies that introduce multiple resolutions through random scale augmentation or independent resolution training, PMSL is designed as a structured coarse-to-fine learning paradigm. The core motivation of PMSL is that thoracic abnormalities in CXR images exhibit substantial scale variations, and lightweight detectors may have difficulty simultaneously capturing global anatomical structures and fine-grained lesion details under fixed-resolution training settings. Therefore, PMSL establishes a progressive feature adaptation pathway, in which the model gradually learns from low-resolution global representations to high-resolution detailed lesion characteristics. Instead of directly optimizing the detector at the highest resolution, PMSL enables hierarchical knowledge acquisition through sequential resolution transitions, thereby improving feature representation capability and small-lesion localization performance.

The PMSL strategy consists of four sequential training stages. The specific progressive schedule was designed according to two considerations: the need to establish stable low-resolution feature representations before introducing high-resolution optimization, and the substantially increased computational cost associated with 1024 × 1024 inputs. The initial 512 × 512 stage provides an efficient starting point for adapting the pretrained detector to CXR-specific anatomical and pathological patterns. A second 512 × 512 stage is retained to consolidate these representations before increasing the spatial resolution, thereby avoiding an abrupt transition from coarse feature learning to computationally intensive high-resolution optimization. The subsequent 640 × 640 stage provides an intermediate resolution for gradual adaptation, while the final 1024 × 1024 stage is used to refine fine-grained representations for small and subtle lesions. Thus, the 512→512→640→1024 schedule was designed as a coarse-to-fine transition with an intermediate stabilization stage rather than as a simple sequence of increasing resolutions. The total optimization budget for PMSL is 200 epochs across all four stages, rather than 200 epochs per stage. Specifically, Stage 1 is trained for 20 epochs, followed by 60 epochs for Stage 2-1, 60 epochs for Stage 2-2, and 60 epochs for Stage 2-3, yielding a total of 200 epochs (20 + 60 + 60 + 60). The shorter initial stage is intended to establish task-specific feature adaptation under the frozen-backbone setting, whereas the subsequent fully trainable stages receive longer optimization periods to progressively refine the detector at increasing resolutions.

In Stage 1, the model is trained at a resolution of 512 × 512, with a training budget of 20 epochs and the first 10 backbone layers frozen (freeze=10). The stage prioritizes rapid learning of thoracic anatomical structures and large-scale abnormalities while adapting the higher-level network components to the CXR detection task. In Stage 2-1, training continues at 512 × 512 resolution for 60 epochs with the entire network unfrozen (freeze=0), allowing further consolidation of the task-specific feature representations established during Stage 1. Subsequently, in Stage 2-2, the input resolution is increased to 640 × 640 for another 60 epochs with freeze=0, enhancing the model's capacity to represent medium-scale lesions. Finally, in Stage 2-3, the resolution is further elevated to 1024 × 1024 for 60 epochs with freeze=0, with a primary focus on improving small-lesion detection sensitivity and fine boundary recognition. Therefore, the four stages receive 20, 60, 60, and 60 epochs, respectively, for a total of 200 training epochs.

The total optimization budget of PMSL-YOLO is therefore 200 epochs across the four sequential stages, rather than 200 epochs per stage. The four stages do not receive equal epoch budgets: Stage 1 accounts for 10% of the total budget, whereas each subsequent stage accounts for 30%. This allocation reflects the progressive optimization strategy, in which the initial frozen-backbone stage provides stable task-specific adaptation at low resolution, while the subsequent fully trainable stages receive longer optimization periods to progressively refine lesion representations as the input resolution increases. The optimization budget is defined in terms of stage-wise epochs rather than an equalized number of parameter-update iterations across stages.

AdamW was used as the optimizer throughout the progressive training process, with cosine annealing applied within each stage. Each subsequent stage was initialized from the best-performing checkpoint obtained from the preceding stage, while Stage 1 was initialized from pretrained YOLOv8n weights. The first 10 backbone layers were frozen in Stage 1 (freeze=10), whereas all layers were unfrozen in the subsequent stages (freeze=0).

The progressive optimization process can be formulated conceptually as a sequential feature refinement process, as expressed in Equation 8:

$$F_{i+1}=Optimization(F_{i},R_{i+1},Y)$$ (8)

Where $F_{i}$ represents the learned feature representation at stage i, $R_{i+1}$ denotes the input resolution of the subsequent stage, and Y represents the corresponding lesion annotations. Through gradually increasing resolution constraints, the model continuously refines feature representations from coarse anatomical information to fine lesion-specific patterns. Compared with direct high-resolution optimization, this progressive feature adaptation strategy reduces the learning difficulty imposed by complex high-resolution representations on lightweight detectors, allowing more stable parameter adaptation and smoother convergence during training.

At the conclusion of each stage, the best-performing checkpoint is selected based exclusively on the performance measured on the fixed validation set described in Section 3.5, and is then used to initialize the subsequent stage. This checkpoint-selection procedure does not involve any gradient updates on validation images. Importantly, the untouched test set remains completely isolated throughout the entire progressive training process: it is never used for gradient-based optimization, hyperparameter tuning, augmentation-parameter selection, checkpoint selection, early-stopping decisions, or stage-to-stage initialization. The test set is accessed only once, after all stage-wise training and model-selection procedures have been finalized, solely for the final independent evaluation. This stage-wise knowledge transfer mechanism reduces the optimization difficulty caused by directly training lightweight models with high-resolution inputs and improves convergence stability. Compared with conventional fixed-resolution training, PMSL provides a more structured learning trajectory for lightweight CXR detectors, allowing the model to achieve a better balance between computational efficiency and multi-scale lesion detection capability. Figure 2 illustrates the proposed PMSL strategy.

**Figure 2:**
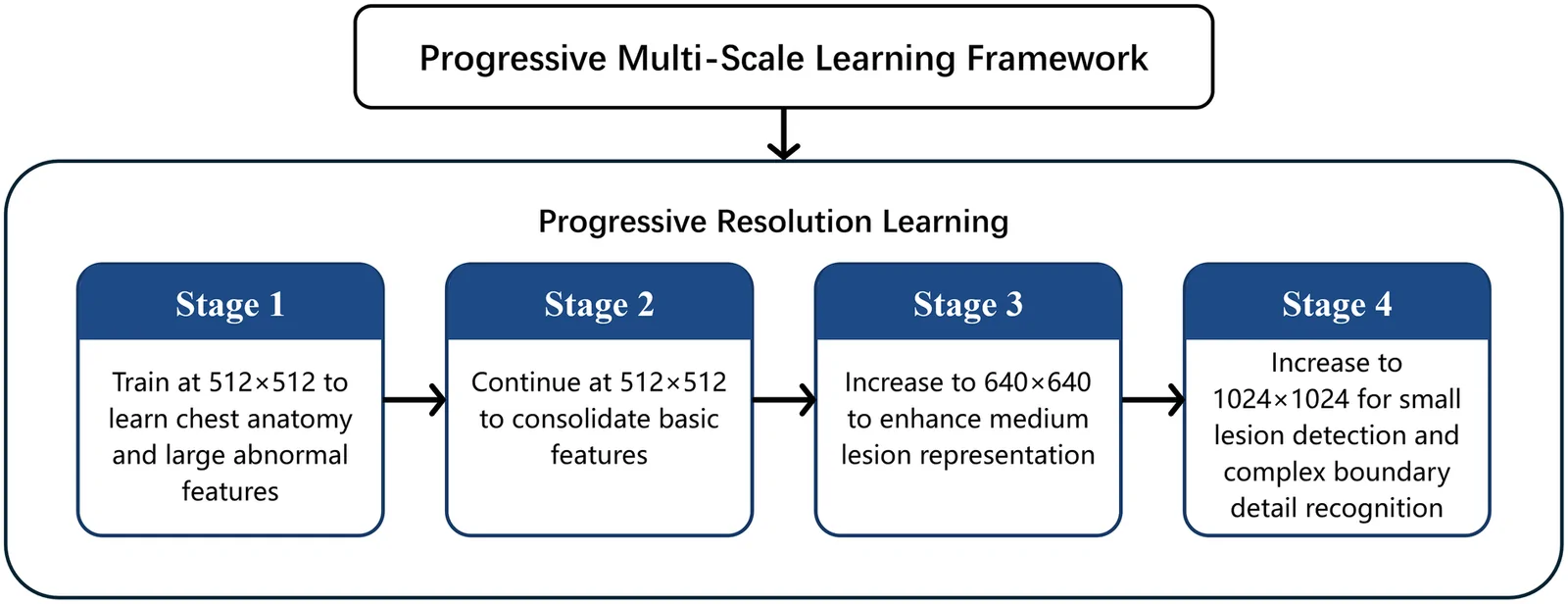
Schematic diagram of the PMSL strategy.

To further examine whether the selected schedule was preferable to an alternative progressive configuration, an additional schedule comparison was conducted under the same dataset split, model architecture, augmentation settings, total training budget of 200 epochs, and random seed. The comparison focused on the proposed 512→512→640→1024 schedule and an alternative 512→640→1024 schedule, in which the intermediate 512 × 512 consolidation stage was removed. This comparison was designed to determine whether retaining the second 512 × 512 stage provides measurable benefits beyond simply increasing the input resolution progressively. The corresponding results are reported in Section 5.3.

Figure 2 illustrates the core mechanism of the proposed PMSL strategy. Instead of directly optimizing a lightweight detector using high-resolution inputs, PMSL adopts a coarse-to-fine learning pathway through sequential resolution transitions. The model first learns global anatomical representations at lower resolution and gradually refines lesion-specific features as the input resolution increases.

### 4.4 Freeze–unfreeze training (FUT) mechanism

To further enhance training stability and feature adaptation during progressive resolution transitions, this study integrates a Freeze–Unfreeze Training (FUT) mechanism into the PMSL strategy. Rather than serving as an independent optimization strategy, FUT is designed as an adaptive parameter updating mechanism that facilitates stable knowledge transfer from pretrained representations to CXR-specific feature representations.

The selection of the freezing depth was motivated by a balance between preserving transferable low-level visual representations and allowing sufficient task-specific adaptation in the higher layers of the network. In the YOLOv8n backbone, early layers primarily capture generic visual features, such as edges, contours, local textures, and basic intensity patterns, which are relatively transferable across image domains. In contrast, deeper backbone layers progressively encode more task-dependent semantic representations. For CXR images, preserving part of the pretrained low-level representations during the initial adaptation stage can therefore reduce unnecessary parameter fluctuations, while keeping the deeper backbone layers, Neck, and Detection Head trainable allows the model to adapt to thoracic anatomical structures and abnormality-specific features.

The freezing depth was further examined empirically by comparing three representative configurations, namely freeze=5, freeze=10, and freeze=15, under the same dataset partition, YOLOv8n architecture, augmentation strategy, optimizer, PMSL schedule, total training budget, and random seed.

The results showed that freeze=10 achieved the best overall detection performance among the three evaluated configurations, suggesting a favorable balance between preserving pretrained representations and enabling task-specific adaptation, as summarized in Table 12.

Therefore, a freezing depth of 10 backbone layers was selected as the default configuration for Stage 1 and consistently applied in the subsequent PMSL experiments.

During the Freeze Phase, which corresponds to Stage 1 of PMSL, the first 10 layers of the Backbone network are frozen (freeze=10), while the remaining trainable components, including the higher-level backbone layers, Neck, and Detection Head, are updated. At this stage, the model focuses on adapting higher-level detection components to the CXR abnormality detection task while preserving general visual representations learned from large-scale pretraining. This stage employs a relatively low input resolution and a smaller learning rate, enabling the model to adapt to the CXR detection task while minimizing early-stage optimization instability.

During the Unfreeze Phase, corresponding to the three sub-stages of Stage 2, the entire network is unfrozen (freeze=0), allowing all backbone layers, the Neck, and the Detection Head to be jointly optimized. With the progressive increase in input resolution from 512 × 512 to 640 × 640 and finally to 1024 × 1024, the model can progressively refine deeper feature representations and improve its ability to capture fine-grained lesion characteristics. Each sub-stage is initialized using the best-performing checkpoint selected from the preceding stage based exclusively on validation-set performance. As emphasized in Sections 3.5 and 4.3, the untouched test set does not participate in any stage-wise checkpoint selection, early-stopping decisions, or model-selection procedures throughout the entire FUT–PMSL training process.

The integration of FUT with PMSL provides a gradual optimization pathway in which the extent of parameter updating is expanded from the initial adaptation stage to full-network optimization. The initial freezing phase preserves transferable pretrained representations and reduces unnecessary parameter fluctuations, whereas subsequent full-network optimization enables the model to adapt its feature hierarchy to increasingly detailed CXR inputs. This strategy reduces the optimization difficulty associated with directly fine-tuning a lightweight detector using high-resolution medical images.

To prevent abrupt parameter fluctuations after unfreezing, differentiated learning-rate settings are applied across stages, while a cosine annealing learning-rate scheduling strategy is consistently maintained throughout the training process.

### 4.5 Evaluation metrics

To comprehensively and objectively evaluate the performance of the proposed PMSL strategy, this study establishes a multi-dimensional evaluation system covering detection accuracy, localization capability, lightweight efficiency, and specialized small-lesion performance.

For detection accuracy, standard object detection metrics are adopted, including mAP@0.5, mAP@0.5:0.95, Precision, and Recall. Specifically, mAP@0.5 reflects comprehensive detection performance under the commonly used IoU threshold of 0.5, while mAP@0.5:0.95 averages performance across multiple IoU thresholds ranging from 0.5 to 0.95 (with a step size of 0.05), providing a more stringent assessment of localization precision.

A clear distinction is maintained between validation-set evaluation and independent test-set evaluation. During model development, the fixed validation set is used solely for monitoring training progress and for selecting the best-performing checkpoint at each PMSL stage. In contrast, the untouched test set is completely withheld from all model-development procedures and is evaluated only once after the final models have been selected, thereby providing an unbiased assessment of generalization performance on previously unseen data.

Given the particular emphasis of this study on small-lesion detection, dedicated statistical analyses are conducted for small-target categories such as Nodule, Infiltration, and Fibrosis. By comparing Precision and Recall across different strategies, the effectiveness of the PMSL approach in enhancing fine-grained lesion detection is specifically validated.

In addition, multiple visualization tools are employed to support performance interpretation, including normalized confusion matrices, Precision–Recall curves, and representative detection case visualizations. These methods facilitate a deeper understanding of model behavior in scenarios involving multi-disease coexistence, small-object detection, and complex anatomical backgrounds.

All evaluation metrics are uniformly computed using the model.val interface provided by Ultralytics YOLOv8, with fixed confidence and IoU threshold settings to ensure fairness, consistency, and reproducibility across all experiments. Validation-set metrics are reported for model-development and comparative analysis, while independent test-set metrics are reported separately to demonstrate generalization performance.

## 5 Experiments and results

### 5.1 Experimental setup

To validate the effectiveness of the proposed lightweight PMSL strategy, this study conducts a series of comparative experiments on the VinBigData CXR Abnormalities Detection dataset. Following the dataset partition and annotation preprocessing protocol detailed in Section 3.5, the retained 15,000 images were divided into 12,000 training, 1,500 validation, and 1,500 untouched test images. The class-wise IoU-based annotation fusion with a threshold of 0.6 was applied consistently before partitioning.

All experiments are implemented using the Ultralytics YOLOv8 framework with official pretrained weights (yolov8n.pt). Baseline512, Baseline1024, and the complete PMSL-YOLO model are evaluated under identical data splits, consistent data augmentation strategies, and a total training budget of 200 epochs. For the single-scale baselines, this corresponds to 200 epochs of continuous training; for PMSL-YOLO, the budget is distributed across the four progressive stages as 20, 60, 60, and 60 epochs, respectively.

Data augmentation is applied exclusively to the training set. The validation and untouched test sets remain completely free of training-time augmentation. For PMSL-YOLO, AdamW with cosine annealing is used within each stage. Stage 1 freezes the first 10 backbone layers, while subsequent stages train the full network. Each stage is initialized from the best validation checkpoint of the preceding stage. The untouched test set is never used for optimization, hyperparameter tuning, checkpoint selection, or stage-to-stage initialization.

All experiments use a fixed random seed to ensure reproducible comparisons within the present protocol. The observed performance differences should therefore be interpreted as empirical results under the specified setting rather than statistically robust estimates across multiple seeds. The experimental platform is summarized in Table 6.

**Table 6:** Experimental environment configuration.

Hardware/Software	Configuration	Description
CPU	Intel Core i9-14900K	24 cores/32 threads
GPU	NVIDIA RTX 5090	32 GB VRAM
Memory	128 GB	Supports large-batch training
Operating System	Ubuntu	–
Framework	Python+PyTorch+YOLOv8	Model training and inference

**Table 7:** Performance comparison of baseline and PMSL-YOLO models on the validation set.

Model	Input Size	Precision (%)	Recall (%)	mAP@0.5 (%)	mAP@0.5:0.95 (%)	Avg Epoch Time (s)
Baseline YOLOv8n	512 × 512	86.7	76.4	81	63.2	5.67
Baseline YOLOv8n	1024 × 1024	88.2	82.5	88.2	67.2	19.62
PMSL-YOLO (Full)	Progressive	95.7	91	91.8	71.2	8.94

### 5.2 Main results

To comprehensively evaluate the effectiveness of the proposed PMSL strategy, this study compares the performance of Baseline512, Baseline1024, and the full PMSL-YOLO model. The primary model-development results are reported on the fixed validation set defined in Section 3.5, which was used for performance monitoring, checkpoint selection, and stage-to-stage model initialization during training. The independent evaluation on the untouched test set is presented separately in Section 5.5.

The experimental results on the validation set demonstrate that the proposed PMSL-YOLO model achieves substantial performance improvements over both baseline configurations. On the validation set, PMSL-YOLO attains an mAP@0.5 of 91.8%, representing an improvement of 10.8 percentage points over Baseline512 and 3.6 percentage points over Baseline1024. The mAP@0.5:0.95 reaches 71.2%, with Precision and Recall of 95.7% and 91.0%, respectively.

Compared with conventional fixed-resolution training strategies, PMSL enables the detector to progressively learn from global anatomical structures to fine-grained lesion representations through a structured coarse-to-fine optimization pathway. This design is particularly suitable for CXR images, where abnormalities usually exhibit substantial variations in size, contrast, and morphological characteristics.

Because the validation set was used for iterative checkpoint selection and stage-to-stage model initialization during progressive training, the validation results should be interpreted as model-development and controlled comparative evidence rather than as an independent estimate of generalization. The independent evaluation conducted on the untouched test set is reported separately in Section 5.5.

It should be noted that the comparison in Table 8 is limited to representative lightweight YOLO-family detectors evaluated under the present experimental protocol and should not be interpreted as a comprehensive comparison with all existing CXR detection methods or as evidence of universal state-of-the-art superiority.

**Table 8:** Comparison with representative lightweight object detectors on the vinBigData CXR validation set.

Model	Input Size	Parameters (M)	FLOPs (G)	Precision (%)	Recall (%)	mAP@0.5 (%)	mAP@0.5:0.95 (%)
YOLOv5n	640 × 640	1.9	4.5	86.1	80.6	84.5	63.8
YOLOv8n	640 × 640	3.2	8.7	86.8	81	85.8	65.3
YOLOv10n	640 × 640	2.3	6.7	88.1	82.5	88.2	67.2
YOLO11n	640 × 640	2.6	6.5	88.6	83.1	88.9	68.1
PMSL-YOLO	Progressive	3.2	22.4	95.7	91	91.8	71.2

For PMSL-YOLO, the reported performance is obtained after progressive multi-scale training. Inference is performed at the final 1024 × 1024 resolution. The reported FLOPs correspond to the final inference resolution.

To further assess the practical deployment efficiency of the proposed PMSL-YOLO, inference latency, throughput, and memory consumption were additionally measured under the same hardware and software environment. The evaluation was performed using the final trained models with a batch size of 1. The measured results are summarized in Table 9.

**Table 9:** Inference efficiency comparison of lightweight CXR detection models.

Model	Input Size	Parameters (M)	FLOPs (G)	Latency (ms/image)	Throughput (FPS)	Peak GPU Memory (MB)
YOLOv8n	512 × 512	3.2	5.5	3.3	300	1,940
YOLOv8n	1024 × 1024	3.2	22.4	8.8	114	3,140
PMSL-YOLO	1024 × 1024	3.2	22.4	8.9	113	3,170

Inference efficiency was evaluated with a batch size of 1 on the NVIDIA RTX 5090 GPU. Inference latency was measured after a GPU warm-up period using repeated forward-pass measurements under the same software environment. Throughput was calculated as FPS=1000/latency (ms/image). Peak GPU memory represents the maximum GPU memory allocated during inference.

As shown in Table 9, PMSL-YOLO introduces no additional trainable parameters or architectural modules compared with the YOLOv8n baseline. Therefore, its inference-time computational complexity remains identical to that of the YOLOv8n architecture at the same 1024 × 1024 input resolution. Although inference at 1024 × 1024 requires higher computational resources than the 512 × 512 baseline, PMSL-YOLO maintains a practical single-image inference latency of 8.9 ms and a throughput of 113 FPS, while requiring 3,170 MB of peak GPU memory under the evaluated hardware environment.

Figure 3 further compares the trends of Recall, mAP@0.5, and mAP@0.5:0.95 across training epochs on the validation set. The PMSL model consistently maintains performance improvements throughout the middle and late stages of training, ultimately converging to a higher performance level, with particularly notable advantages in Recall. These validation curves were used for performance monitoring and stage-wise checkpoint selection during model development; therefore, the observed trends should be interpreted as evidence of optimization behavior within the present validation protocol rather than as independent evidence of generalization to unseen data.

**Figure 3:**
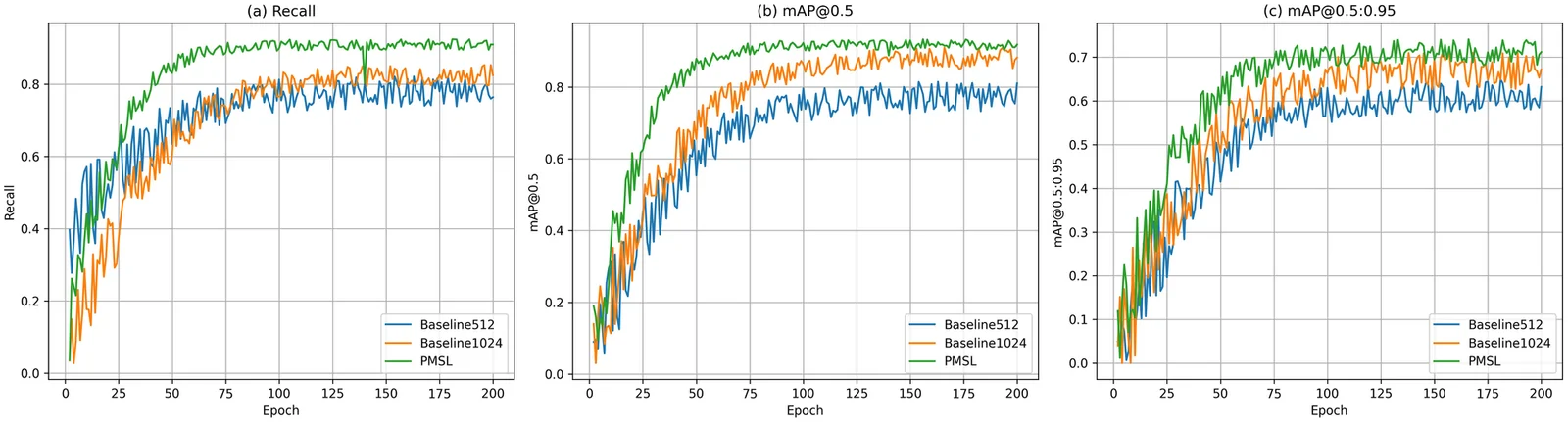
Validation performance curves of baseline and PMSL models. (a) Recall; (b) mAP@0.5; (c) 2 mAP@0.5:0.95.

To visually demonstrate qualitative detection behavior, Figure 4 presents representative detection examples. The proposed framework correctly identifies multiple thoracic abnormalities and shows visually consistent localization in cases involving blurred lesion boundaries and overlapping multi-lesion patterns.

**Figure 4:**
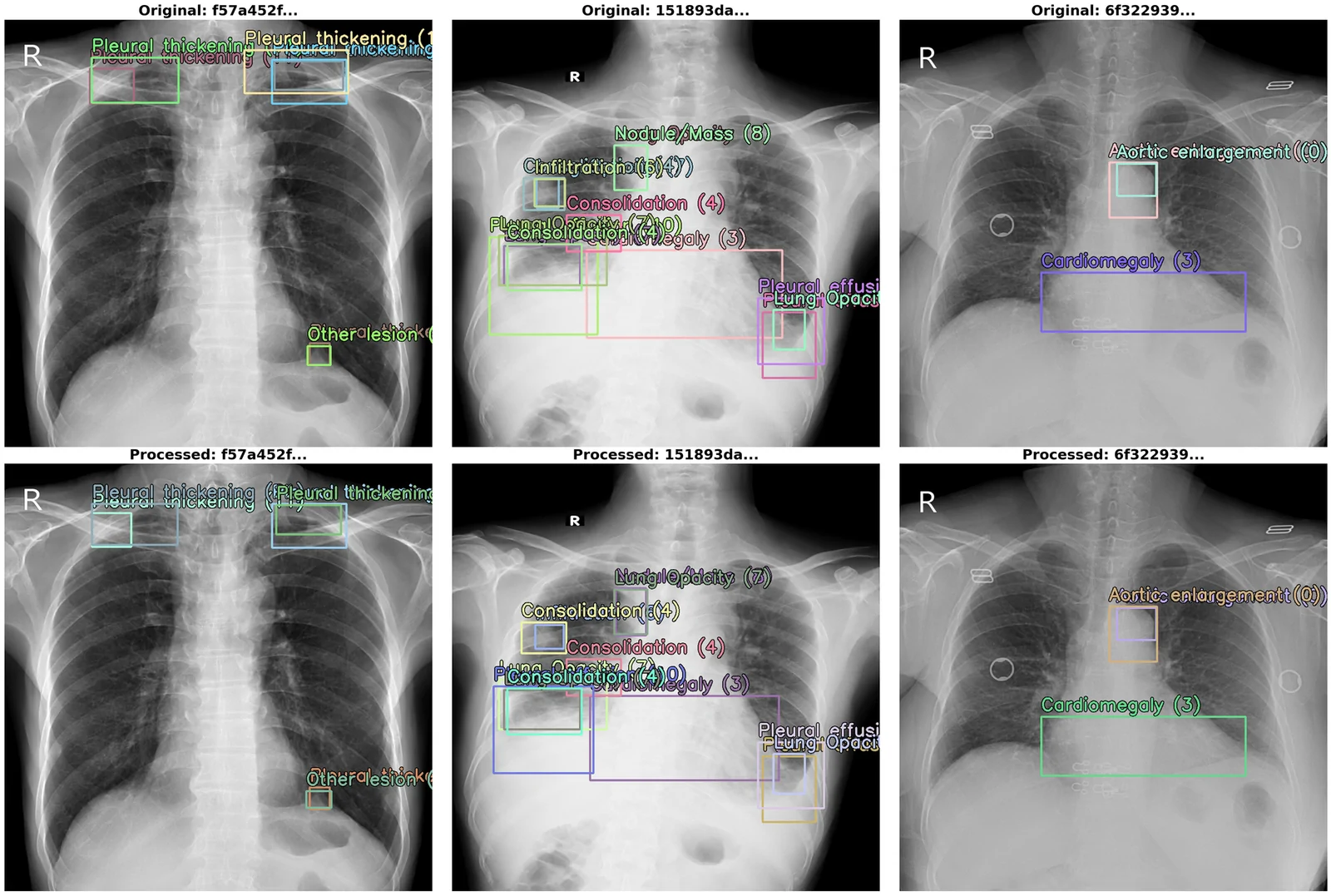
Representative detection examples on the validation set.

The experimental findings provide empirical evidence supporting the effectiveness of the PMSL strategy. While preserving the lightweight advantages of YOLOv8n, PMSL substantially improves detection accuracy, recall, and sensitivity to ambiguous lesion boundaries. The validation-based comparisons provide controlled evidence of the performance gains achieved during model development, while the independent evaluation on the untouched test set is presented separately in Section 5.5 to assess whether these performance gains are also observed on data that remained completely unseen during model development.

### 5.3 Ablation studies

To systematically evaluate the contribution of each proposed component and investigate the underlying mechanism of performance improvement, comprehensive ablation experiments are conducted. The analysis focuses on three key components: the data augmentation strategy, the PMSL strategy, and the FUT mechanism. All ablation experiments are performed using identical dataset partitions, training epochs of 200, optimizer configurations, and random seed settings (seed=42) to ensure fair and reproducible comparisons. Unlike the main performance comparison experiments, the ablation study aims to isolate the individual effect of each component and evaluate its specific contribution to the final PMSL strategy. Table 10 summarizes the performance variations under different module combinations.

**Table 10:** Ablation study results.

Method	Precision	Recall	mAP@0.5	mAP@0.5:0.95	Avg Epoch Time (s)	Description
Baseline YOLOv8n (512)	86.7	76.4	81	63.2	5.58	Single-scale training without freezing or augmentation
Baseline YOLOv8n (1024)	88.2	82.5	88.2	67.2	19.12	Direct high-resolution single-scale training
+ Freeze	87.1	79.6	81.7	63.4	4.7	Backbone freezing strategy applied
+ Aug	88.2	81.8	83.5	66.7	4.77	Enhanced data augmentation applied
+ Progressive	90.5	83.5	88.5	69.2	8.68	Progressive multi-scale learning strategy
+ Full PMSL	95.7	91	91.8	71.2	9.69	Progressive multi-scale learning with LR optimization

All experiments are conducted under the same hardware environment and dataset partition.

As model-development experiments, all ablation variants were evaluated using the same fixed validation set, while the untouched test set was excluded from ablation-based model selection and comparison. This protocol ensures a consistent basis for internal comparison without using the test set to guide model-development decisions.

The ablation results demonstrate that the proposed components contribute differently to the overall performance. Among the evaluated components, the progressive multi-scale learning strategy provides the largest individual improvement in mAP and Recall, supporting the interpretation that progressive resolution adaptation is a major contributor to the observed improvement in lesion representation capability.

To further isolate the contribution of progressive resolution adaptation, the “+ Progressive” setting removes the Freeze–Unfreeze mechanism and retains the original augmentation strategy, enabling a direct evaluation of PMSL itself. The Freeze–Unfreeze mechanism mainly improves optimization stability during transfer learning, while the CXR-specific augmentation strategy contributes to improved robustness under variations in lesion appearance and image quality. The final “Full PMSL” configuration additionally incorporates staged optimization with learning-rate adjustment across different resolution stages, leading to further performance gains compared with progressive training alone.

#### 5.3.1 Ablation analysis of the data augmentation strategy

When advanced augmentation techniques such as Mosaic and MixUp are removed, both mAP@0.5 and Recall decrease noticeably. This indicates that appropriate augmentation strategies are beneficial for addressing the inherent challenges of CXR datasets, including lesion scale variation, limited abnormal samples, and imaging quality differences. However, augmentation alone cannot fully compensate for the limitations of fixed-resolution feature learning, highlighting the importance of integrating augmentation with the proposed PMSL strategy.

#### 5.3.2 Ablation analysis of the PMSL strategy

This study compares single-scale training, progressive resolution training, and the complete four-stage PMSL strategy. As illustrated in Figure 5, model performance generally improves along the progressive training trajectory as the input resolution transitions from the initial 512 × 512 stage to 640 × 640 and finally to 1024 × 1024. The results indicate that staged resolution adaptation progressively enhances the model's ability to learn representations at different spatial scales, providing additional feature learning benefits compared with direct single-resolution training. In particular, the progressive transition toward higher resolutions contributes to the refinement of fine-grained lesion representations and localization capability, which is particularly relevant to small and subtle abnormalities in CXR images.

**Figure 5:**
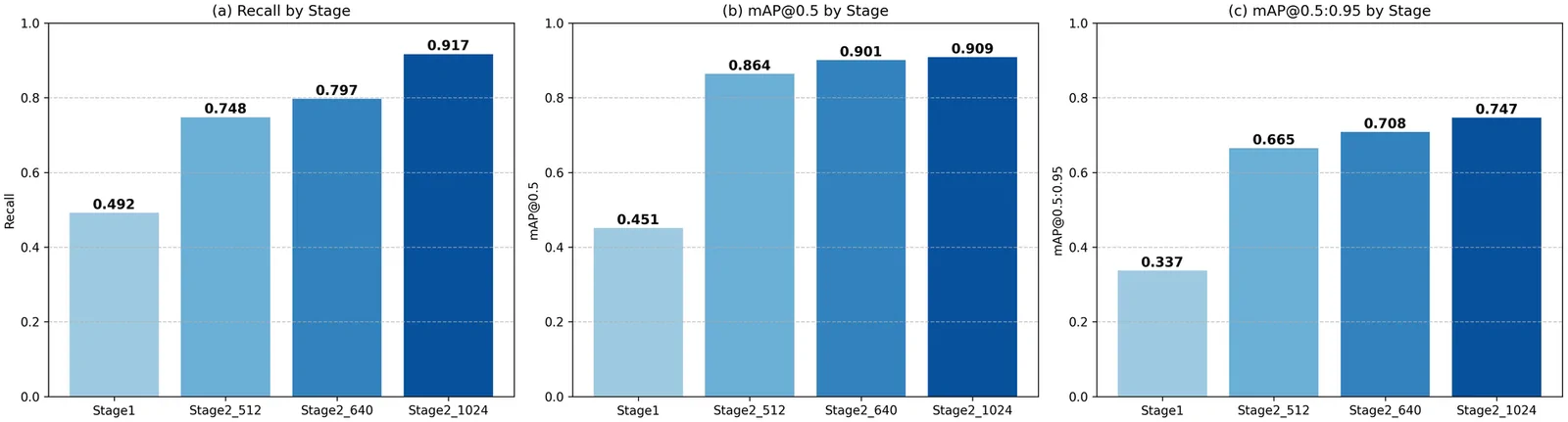
Performance comparison across different PMSL training stages. (a) Recall by stage; (b) 2 mAP@0.5 by stage; (c) mAP@0.5:0.95 by stage.

The results support the underlying design principle of PMSL: low-resolution stages encourage efficient learning of global anatomical structures, whereas subsequent higher-resolution stages gradually enhance fine-grained lesion representation and boundary localization capability.

#### 5.3.3 Sensitivity analysis of the progressive schedule

To further justify the specific resolution schedule adopted in PMSL, an additional schedule-sensitivity experiment was conducted using an alternative progressive configuration. The proposed schedule was compared with a simplified progressive schedule, in which the intermediate 512 × 512 consolidation stage was removed. This comparison was designed to determine whether the additional 512 × 512 stage provides measurable benefits beyond a conventional monotonic increase in input resolution.

To ensure a controlled comparison, both schedules used the same YOLOv8n architecture, dataset partition, annotation preprocessing, data augmentation strategy, optimizer, random seed, and overall training budget. For both schedules, the total training budget was fixed at 200 epochs, and the same training budget was used for the comparative evaluation. Therefore, the primary experimental difference was the resolution-transition schedule itself.

This schedule-sensitivity analysis was conducted independently of the component ablation reported in Table 10 and was specifically designed to evaluate the effect of the resolution-transition schedule under the complete PMSL training configuration. The comparison therefore provides an empirical assessment of whether retaining the intermediate 512 × 512 consolidation stage contributes to the final detection performance. The results are summarized in Table 11.

**Table 11:** Sensitivity analysis of the PMSL progressive training schedule.

Schedule	Precision (%)	Recall (%)	mAP@0.5 (%)	mAP@0.5:0.95 (%)	Avg. Epoch Time (s)
512→640→1024	94.4	89.5	90.4	70.1	9.18
512→512→640→1024	95.7	91.0	91.8	71.2	9.75

As shown in Table 11, the proposed 512→512→640→1024 schedule achieves 95.7% Precision, 91.0% Recall, 91.8% mAP@0.5, and 71.2% mAP@0.5:0.95, compared with 94.4%, 89.5%, 90.4%, and 70.1%, respectively, for the simplified 512→640→1024 schedule. The proposed schedule improves mAP@0.5 by 1.4 percentage points and mAP@0.5:0.95 by 1.1 percentage points, while also improving Precision from 94.4% to 95.7% and Recall from 89.5% to 91.0%.

These results indicate that retaining the second 512 × 512 stage provides additional performance benefits before the model transitions to higher-resolution optimization. The findings support the interpretation that the intermediate 512 × 512 consolidation stage facilitates a more stable transition from initial low-resolution adaptation to subsequent high-resolution optimization. Therefore, the selected 512→512→640→1024 schedule is empirically supported under the experimental conditions of this study. Nevertheless, this comparison does not imply that the proposed schedule is universally optimal. Other resolution combinations or stage durations may be beneficial under different datasets, model architectures, or computational constraints.

#### 5.3.4 Sensitivity analysis of the freeze depth

To further justify the selection of the freezing depth in the FUT mechanism, an additional sensitivity analysis was conducted by varying the number of frozen backbone layers during the initial training stage. Three representative configurations were evaluated under the same dataset partition, annotation preprocessing procedure, augmentation strategy, optimizer, training budget, and random seed. All other PMSL configurations, including the progressive resolution schedule, were kept identical, such that the freeze depth was the only experimental factor varied in this analysis.

The purpose of this experiment was to determine whether the selected freezing depth provides an appropriate balance between preserving transferable pretrained representations and allowing sufficient adaptation to CXR-specific visual characteristics. A smaller freezing depth permits more backbone parameters to adapt during the initial stage, whereas a larger freezing depth preserves more pretrained representations but may restrict task-specific adaptation. The results are summarized in Table 12.

**Table 12:** Sensitivity analysis of the freeze depth in the FUT mechanism.

Freeze Depth	Precision (%)	Recall (%)	mAP@0.5 (%)	mAP@0.5:0.95 (%)	Avg. Epoch Time (s)
5	93.7	87.3	89.5	69.2	8.69
10	94.7	89	91.6	70.5	9.74
15	94	88.8	90.7	69.9	10.82

Results were obtained from independent runs under the complete PMSL schedule with only the freeze depth varied. Minor numerical differences from the main results in Table 7 are attributable to independent optimization trajectories.

The freeze=10 configuration achieves the highest performance among the three evaluated settings, reaching 94.7% Precision, 89.0% Recall, 91.6% mAP@0.5, and 70.5% mAP@0.5:0.95. Compared with freeze=5, it improves Recall by 1.7 percentage points, mAP@0.5 by 2.1 percentage points, and mAP@0.5:0.95 by 1.3 percentage points. Compared with freeze=15, the improvements are 0.2, 0.9, and 0.6 percentage points, respectively. These results indicate that freeze=10 provides the most favorable performance among the three evaluated freezing depths. The absolute values differ slightly from the main experimental results because each freeze-depth setting was evaluated in an independent run; the relative ranking remains consistent.

The results indicate that a smaller freezing depth does not necessarily provide better task adaptation, as allowing more backbone parameters to update during the initial stage may reduce the benefit of retaining transferable pretrained representations. Conversely, a larger freezing depth preserves more pretrained parameters but restricts the extent of early task-specific adaptation. The intermediate freeze=10 configuration therefore provides a more favorable balance between representation preservation and CXR-specific feature adaptation under the experimental conditions of this study.

Accordingly, freeze=10 was selected for Stage 1 and consistently applied in the subsequent PMSL experiments. This selection is empirically supported by the controlled freeze-depth sensitivity analysis rather than being based solely on an *a priori* architectural assumption.

#### 5.3.5 Ablation analysis of the FUT mechanism

Comparative experiments between full-parameter direct training and staged FUT show qualitatively smoother loss curves during the early training stages and an earlier apparent convergence trend in the present training run. These results suggest that FUT facilitates more stable knowledge transfer from pretrained weights by gradually adapting feature extraction layers to the characteristics of CXR images. Furthermore, the freeze-depth sensitivity analysis in Table 12 demonstrates that the freeze=10 configuration achieves the highest detection performance among the evaluated freezing depths, providing empirical support for its use during the initial adaptation stage. This mechanism is particularly beneficial when lightweight models are fine-tuned on medical datasets with limited training samples.

#### 5.3.6 Ablation analysis of the explainability module

Explainability modules such as Grad-CAM have minimal direct impact on detection accuracy metrics, although they introduce slight increases in inference time. Therefore, the explainability module is considered an auxiliary interpretability component rather than a performance-enhancing component. In this study, Grad-CAM is used as a *post-hoc* visualization tool to qualitatively inspect the spatial regions associated with model predictions and to support model behavior analysis and error analysis. However, these visualizations are not intended to constitute clinically validated explanations or to establish the faithfulness or localization accuracy of the model's internal decision process.

### 5.4 Small lesion detection performance analysis

Small lesion detection represents one of the most critical and challenging tasks in intelligent CXR analysis. Due to the ambiguous boundaries and heterogeneous scales of CXR abnormalities, conventional pixel-based small-object definitions may not fully reflect clinical lesion characteristics. This study adopts category-level AP analysis as a complementary evaluation strategy for representative small-lesion categories. Subtle abnormalities such as pulmonary nodules, focal infiltrations, and limited consolidations are typically characterized by small size, blurred boundaries, and low contrast, making them highly susceptible to missed detection. Therefore, beyond overall detection metrics, a dedicated analysis focusing on small-lesion categories is necessary to further evaluate the capability of PMSL-YOLO in capturing fine-grained abnormal patterns.

To objectively evaluate the performance of PMSL-YOLO in small lesion detection tasks, this study conducts a targeted analysis of representative small-lesion categories. Specifically, categories with relatively small lesion characteristics, including Nodule, Infiltration, and Consolidation, are selected for detailed evaluation based on detection results on the validation set. The results are summarized in Table 13.

**Table 13:** Detection performance of representative small-lesion categories.

Lesion Category	TP	Total Samples	Recall (%)	Major Misclassifications
Nodule	214	256	83.6	Calcification, Other lesion, Lung Opacity
Infiltration	108	125	86.4	Consolidation, Lung Opacity
Consolidation	46	56	82.1	Infiltration, Lung Opacity

As shown in Table 13, PMSL-YOLO achieves consistently high Recall across all three representative small-lesion categories. For pulmonary nodules, the model attains a Recall of 83.6%, correctly identifying 214 out of 256 ground-truth instances. Misclassifications are primarily concentrated in calcification, other lesion, and lung opacity, which may be associated with the visual overlap between small nodular opacities and other focal pulmonary abnormalities. For the infiltration category, Recall reaches 86.4%, with 108 out of 125 ground-truth instances correctly detected; the major misclassifications occur as consolidation or lung opacity. Consolidation achieves a Recall of 82.1%, with 46 out of 56 ground-truth instances correctly detected, similarly indicating relatively stable detection performance across the three representative categories.

To provide a more comprehensive evaluation of small-lesion detection capability, category-level AP analysis was additionally performed for representative lesion categories. Unlike conventional object detection tasks where small objects are usually defined according to pixel-level bounding box size, CXR abnormalities exhibit large variations in scale and ambiguous lesion boundaries. This study adopts disease-category AP analysis for representative small-lesion categories as a complementary evaluation approach. The corresponding category-level AP results are summarized in Table 14.

**Table 14:** Category-level AP comparison for representative small-lesion categories.

Model	Nodule AP(%)	Infiltration AP(%)	Consolidation AP(%)	Mean AP(%)
Baseline YOLOv8n (512 × 512)	64.7	72.1	67.6	68
Baseline YOLOv8n (1024 × 1024)	71.4	77.4	74.3	74.5
PMSL-YOLO (Progressive 512→512→640→1024)	78.3	83.3	80.8	80.9

The results in Table 14 demonstrate that PMSL-YOLO consistently improves AP across all representative small-lesion categories. Compared with Baseline YOLOv8n (512 × 512), PMSL-YOLO improves Nodule AP from 64.7% to 78.3%, Infiltration AP from 72.1% to 83.3%, and Consolidation AP from 67.6% to 80.8%. The corresponding mean AP increases from 68.0% to 80.9%.

These improvements suggest that progressive multi-scale learning facilitates more effective representation of subtle lesion patterns, particularly for abnormalities with limited spatial extent and weak contrast. Compared with direct high-resolution training (Baseline YOLOv8n, 1024 × 1024), PMSL-YOLO also achieves higher category-level AP, with improvements of 6.9, 5.9, and 6.5 percentage points for Nodule, Infiltration, and Consolidation, respectively. This comparison suggests that the observed gains are not explained solely by increased input resolution. The results are consistent with the intended role of PMSL in progressively adapting feature representations across multiple resolutions, although further controlled studies would be required to isolate the contribution of progressive feature adaptation from other training factors.

Error analysis based on the detection results indicates that the remaining errors of PMSL-YOLO are predominantly characterized by misclassification among visually similar disease categories rather than complete omission of lesion regions. This pattern suggests that a substantial proportion of the residual errors may be associated with intrinsic radiographic similarity between disease categories. From a clinical diagnostic assistance perspective, the detected suspicious regions may provide useful visual cues for further review by radiologists together with additional clinical information.

To more intuitively evaluate the qualitative differences among different training strategies for small lesion detection in CXR, representative validation cases are selected for visual comparative analysis among Baseline YOLOv8n (512 × 512), Baseline YOLOv8n (1024 × 1024), and the final PMSL-YOLO model trained under the progressive multi-scale framework. Particular attention is given to lesion localization capability, bounding box quality, and missed detection rates for subtle abnormalities such as pulmonary nodules, focal infiltrations, and limited consolidations.

Figure 6 presents the comparative visualization of detection results across different training strategies on identical CXR images.

**Figure 6:**
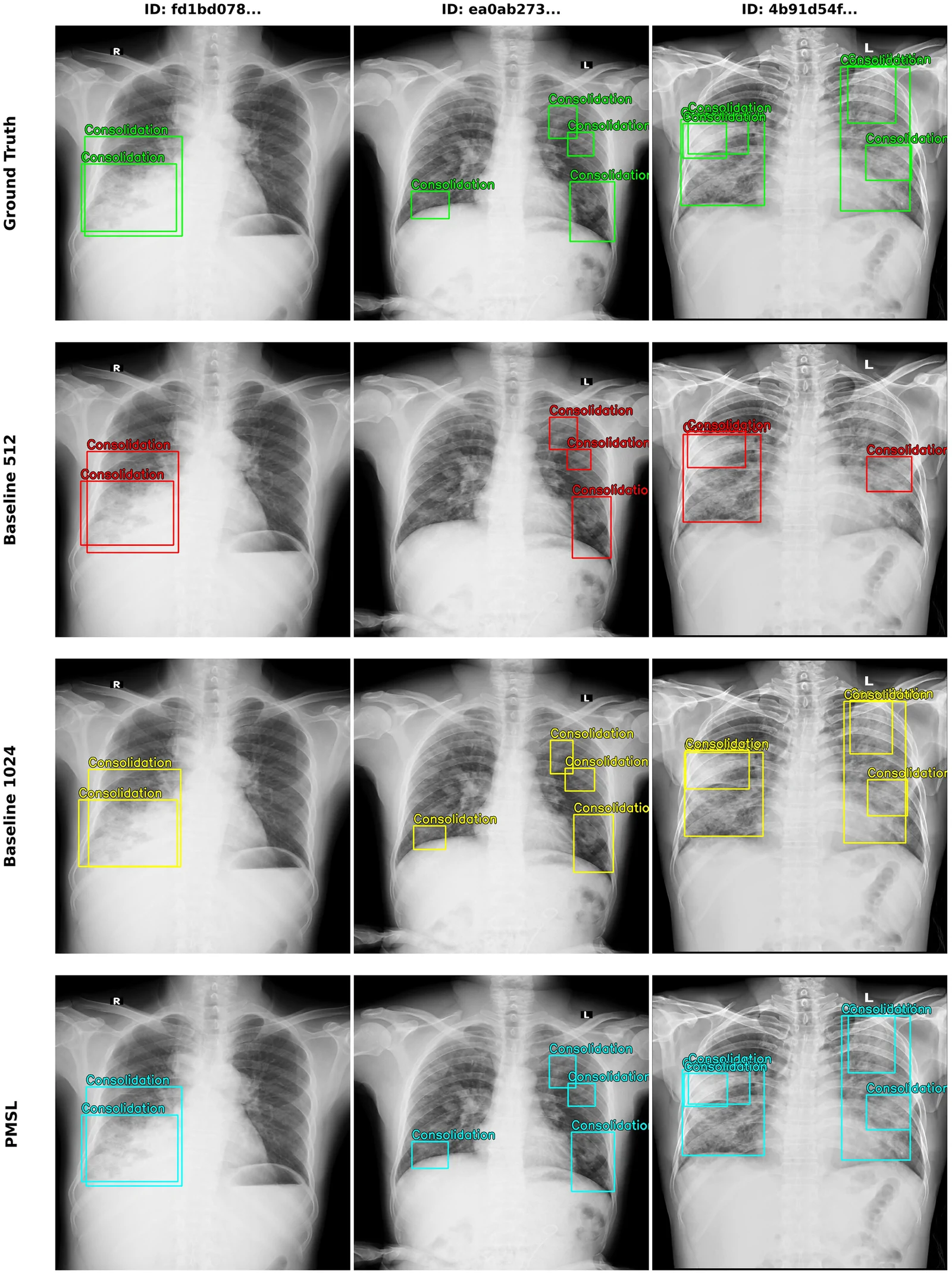
Visual comparison of small lesion detection under different training strategies.

The qualitative results in the illustrated validation cases indicate that Baseline512 exhibits more apparent missed detections and boundary misalignment for small lesions in the selected cases. Although Baseline1024 shows some improvement, some localization instability remains in the illustrated cases, despite its substantially higher computational cost. In contrast, PMSL-YOLO shows more complete localization of subtle lesion regions in these representative cases, with fewer apparent missed detections and improved bounding-box coverage. These qualitative observations are intended to complement, rather than replace, the quantitative evaluation reported in Tables 13, 14.

To further elucidate the underlying mechanism of PMSL, this study analyzes the evolutionary trend of Recall for representative small lesions throughout the four-stage training process. The results are illustrated in Figure 7.

**Figure 7:**
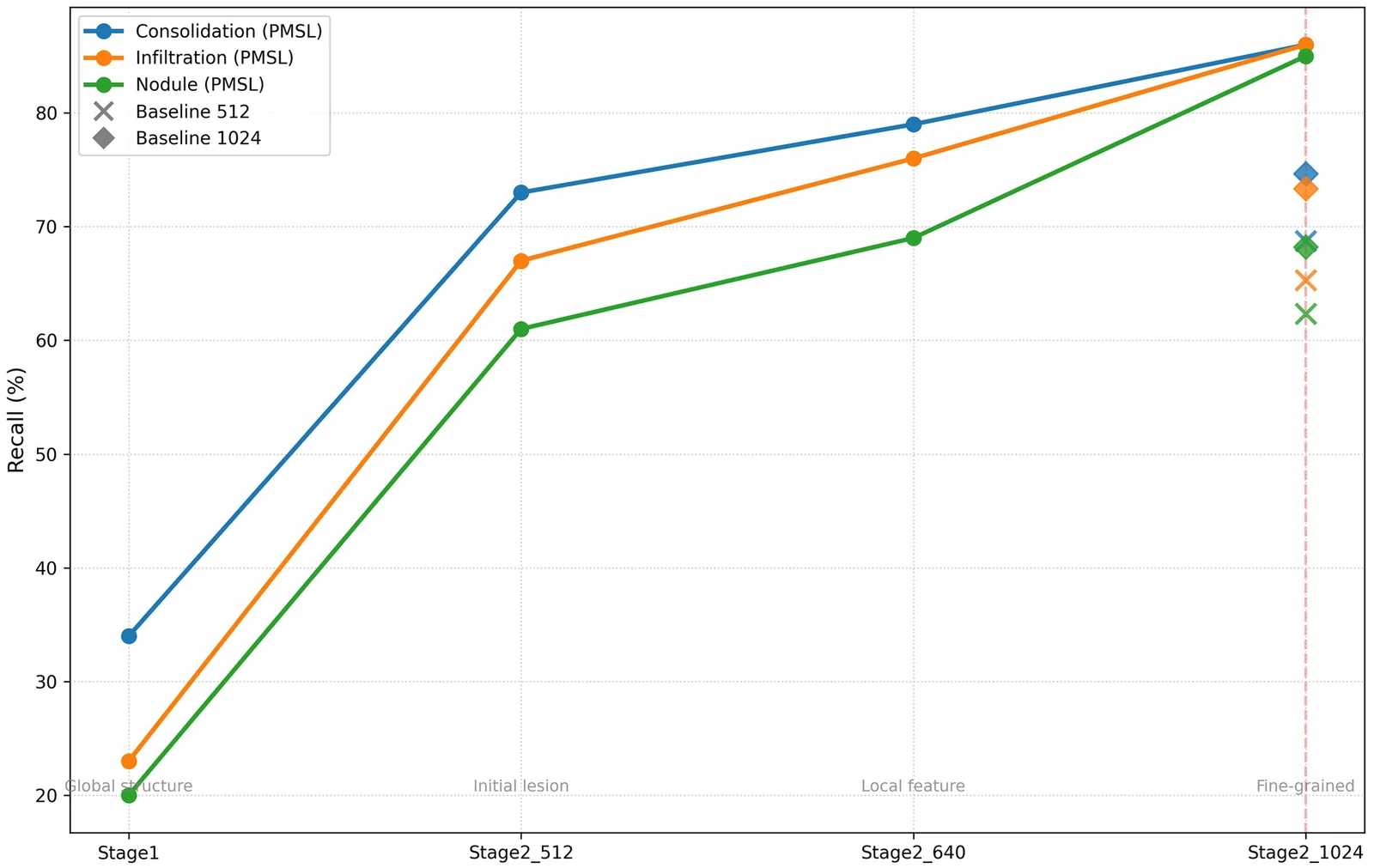
Evolution of small-lesion recall across PMSL training stages.

The results demonstrate a consistent stage-wise increase in Recall for all three representative small-lesion categories throughout PMSL-YOLO training. Stage 1 yields relatively low initial Recall, with values of 0.34 for Consolidation, 0.23 for Infiltration, and 0.20 for Nodule. During Stage 2-512, Recall increases substantially to 0.73, 0.67, and 0.61, respectively. In Stage 2-640, further increases are observed, reaching 0.79, 0.76, and 0.69. Finally, Stage 2-1024 achieves the highest Recall values of 0.821 for Consolidation, 0.864 for Infiltration, and 0.836 for Nodule, which are consistent with the category-level results reported in Table 13. The consistent improvement across all three categories indicates that the successive training stages progressively enhance the model's ability to detect representative small lesions.

Compared with the baseline models, simply increasing the input resolution improves small-lesion detection performance, but the resulting performance remains below that achieved by the complete four-stage PMSL strategy in the present experiments. Together with the stage-wise Recall evolution shown in Figure 9, these results suggest that the performance improvement is not solely attributable to higher-resolution input, but is also associated with the progressive multi-scale optimization process introduced by PMSL.

In summary, the dedicated small-lesion analysis provides empirical evidence supporting the effectiveness of PMSL-YOLO from multiple complementary perspectives, including category-level AP analysis, error pattern analysis, qualitative visualization, and stage-wise performance evolution on the fixed validation set. The results suggest that PMSL provides an effective strategy for improving sensitivity to subtle thoracic abnormalities while maintaining the lightweight characteristics of YOLOv8n. These findings represent model-development and comparative evidence obtained under the present VinBigData dataset and experimental protocol rather than an independent estimate of clinical generalization. Nevertheless, further validation on larger multi-center datasets, cross-device data, and prospective clinical scenarios is required before real-world deployment.

### 5.5 Independent test evaluation

To further assess generalization and mitigate model-selection bias from repeated validation-set use, a completely untouched 1,500-image test set was reserved and never used for training, augmentation, hyperparameter tuning, checkpoint selection, or stage-to-stage initialization. Baseline YOLOv8n and PMSL-YOLO were evaluated on this set. Results are summarized in Table 15.

**Table 15:** Independent evaluation on the untouched test set.

Model	Input Size	Precision (%)	Recall (%)	mAP@0.5 (%)	mAP@0.5:0.95 (%)
Baseline YOLOv8n	512 × 512	85.4	77.8	82.1	62
Baseline YOLOv8n	1024 × 1024	89.6	80.9	86.7	68.4
PMSL-YOLO	Progressive (512→512→640→1024)	94.1	92.4	93.3	70.1

On the untouched test set, PMSL-YOLO achieves 94.1% Precision, 92.4% Recall, 93.3% mAP@0.5, and 70.1% mAP@0.5:0.95. Compared with Baseline YOLOv8n at 512 × 512, PMSL-YOLO improves mAP@0.5 and mAP@0.5:0.95 by 11.2 and 8.1 percentage points, respectively. Compared with the 1024 × 1024 baseline, the corresponding improvements are 6.6 and 1.7 percentage points. In addition, PMSL-YOLO achieves higher Precision and Recall than both baseline configurations, with improvements of 8.7 and 14.6 percentage points over the 512 × 512 baseline, and 4.5 and 11.5 percentage points over the 1024 × 1024 baseline, respectively.

The independent test results also provide a useful comparison with the validation-set results reported in the preceding experiments. Although the absolute values differ across the two subsets, PMSL-YOLO maintains its performance advantage over both baseline configurations on the untouched test set. In particular, the test-set mAP@0.5 of 93.3% remains close to the corresponding validation-set performance, while the test-set mAP@0.5:0.95 of 70.1% is also comparable to the validation results. This consistency suggests that the performance improvements observed during model development are not restricted to the repeatedly monitored validation subset and are also observed on the independently reserved test subset. These results therefore provide an independent check on the robustness of the model-selection process and offer additional evidence that the observed performance improvements are maintained under the present dataset and experimental protocol.

It should be noted that this evaluation represents an independent test within the same VinBigData dataset rather than external validation across different institutions, imaging devices, or patient populations. Therefore, the results support the reproducibility of the observed performance improvements on the reserved test subset, but do not by themselves establish broader clinical generalization. Further evaluation on external multi-center datasets would be required to assess cross-domain and real-world clinical generalization.

## 6 Discussion

### 6.1 Principal findings

This study develops a lightweight CXR multi-disease detection framework based on YOLOv8n and achieves a favorable balance among detection performance, training efficiency, and deployment feasibility through PMSL, the FUT mechanism, and medical-image-oriented data augmentation. The main validation-set experiments demonstrate substantial improvements over fixed-resolution baselines, while evaluation on the completely untouched test set shows that these gains are also observed on data not used during model development. On the independent test set, PMSL-YOLO with the progressive 512→512→640→1024 configuration achieves 94.1% Precision, 92.4% Recall, 93.3% mAP@0.5, and 70.1% mAP@0.5:0.95, compared with 89.6%, 80.9%, 86.7%, and 68.4%, respectively, for the 1024 × 1024 baseline. Because all experiments were conducted with a single random seed, these improvements should not be interpreted as statistically robust estimates across repeated training runs.

Experimental results demonstrate that PMSL improves the handling of scale-variant lesions, with particularly clear benefits for small-lesion detection. By progressively increasing the input resolution, the model can transition from learning global anatomical representations to refining fine-grained lesion features. The ablation and schedule-sensitivity analyses support the contribution of progressive resolution adaptation beyond simply increasing the input resolution, while the FUT mechanism and medical-image-oriented augmentation provide complementary contributions to the overall performance.

The ablation results further indicate that PMSL is the primary contributor to the observed performance improvement, whereas FUT mainly supports parameter adaptation during resolution transitions. The schedule-sensitivity results also indicate that the resolution-transition configuration affects the final performance, highlighting the importance of the progressive training design. Importantly, the independent test evaluation provides additional evidence that the performance gains are observed beyond the validation subset used for model development.

The findings suggest that PMSL can improve detection performance without increasing the parameter count or introducing additional architectural modules. Its potential advantage therefore lies in progressive optimization of a lightweight detector rather than increased model capacity, although broader external generalization and repeated-run robustness remain to be established.

### 6.2 Clinical and practical implications

The proposed lightweight PMSL strategy shows potential clinical and practical value for future CXR diagnostic assistance. The performance improvements observed on the validation set are also observed on the completely untouched test subset, providing independent evaluation within the same VinBigData dataset. However, the current evidence does not establish generalization across independent institutions, imaging devices, patient populations, or clinical workflows. The framework should therefore be considered a potential decision-support tool rather than a clinically validated diagnostic system.

First, the framework may support more efficient CXR interpretation by providing rapid multi-disease detection, lesion locations, categories, and confidence scores. Its lightweight architecture and measured inference efficiency indicate potential suitability for applications with constrained computational resources or response-time requirements. Such applications may include primary healthcare and telemedicine, although actual clinical utility requires prospective evaluation under real-world workflow conditions.

Second, the improved detection of representative small-lesion categories may have practical relevance for identifying subtle thoracic abnormalities. The small-lesion analysis and stage-wise Recall results provide complementary evidence of improved detection across Nodule, Infiltration, and Consolidation categories. However, whether these improvements translate into earlier disease detection, improved diagnostic decisions, or better patient outcomes remains to be established through prospective clinical studies.

The model is intended to assist rather than replace radiologists by providing additional quantitative and visual information. Grad-CAM visualizations should be regarded as qualitative, *post-hoc* model visualizations rather than clinically validated explanations. Similarly, the transferability of PMSL to other imaging modalities such as CT, mammography, or ultrasound remains to be empirically evaluated because of differences in imaging characteristics and lesion distributions.

### 6.3 Advantages of PMSL

PMSL is a key methodological contribution of this study. Rather than simply increasing the input resolution, it establishes a structured coarse-to-fine training pathway in which the model progressively transitions from global structural representation learning to fine-grained lesion refinement. The schedule-sensitivity analysis indicates that the resolution-transition configuration affects performance, supporting the importance of this progressive design.

Compared with fixed-resolution training, PMSL shows clear benefits for small-lesion detection under the present experimental conditions. The lower-resolution stages provide an initial basis for learning global thoracic structures, while subsequent higher-resolution stages enable further refinement of subtle lesion boundaries and local features. The category-level AP and stage-wise Recall analyses provide complementary evidence for this progressive improvement.

A further advantage is the integration of PMSL with the Freeze–Unfreeze mechanism. By carrying forward optimized weights across resolution transitions, the model can progressively adapt its parameters as the input resolution increases. The ablation results indicate that FUT contributes to stable parameter adaptation, while PMSL remains the primary contributor to the observed performance gains. At the same time, PMSL does not introduce additional trainable parameters or architectural modules, preserving the lightweight YOLOv8n structure.

Therefore, the main advantage of PMSL lies in progressive refinement of a lightweight detector through structured resolution transitions rather than increased model capacity. The independent test results provide additional evidence that the resulting performance gains are also observed on the reserved test subset. Nevertheless, the current evidence is limited to the present dataset and experimental conditions, and further repeated-seed and external multi-center evaluations are needed to establish broader robustness and generalizability.

### 6.4 Limitations and future work

Although the proposed PMSL strategy achieves promising performance in detection accuracy, model efficiency, and small-lesion recognition, several limitations remain.

First, the present study relies on the VinBigData dataset as a single data source. Although the dataset provides a relatively large and consistently annotated collection of chest X-rays, the current evaluation does not include external datasets or cross-institutional and cross-device validation. Therefore, the generalizability of PMSL-YOLO to different patient populations, imaging devices, acquisition protocols, and healthcare settings remains to be established. Future work should conduct multi-center and external validation to assess the robustness of the proposed framework under heterogeneous real-world conditions.

Second, the current experiments use a single random seed (42) to ensure controlled and reproducible comparisons. While this setting reduces experimental variability within the present study, it does not characterize the variability of model performance across different random initializations. Future studies should perform repeated experiments with multiple random seeds and report mean performance together with variance or confidence estimates to further assess statistical robustness.

Third, the model still exhibits occasional false negatives and misclassifications in extremely small lesions or low-contrast regions. These errors indicate that further improvements in fine-grained feature representation may be beneficial. Future research could investigate lightweight feature-enhancement, attention-based, or Transformer-assisted approaches while preserving the computational advantages of the proposed framework.

Fourth, the current explainability analysis mainly relies on *post-hoc* Grad-CAM visualization, which provides qualitative localization cues but does not constitute a clinically validated explanation of model reasoning. Future work could explore more clinically aligned explainability methods, including attention-guided representations, prototype-based approaches, or medical knowledge–driven reasoning.

Finally, the present evaluation remains retrospective and dataset-based, without prospective assessment in real clinical workflows. Although the independent test evaluation provides evidence of generalization within the same dataset and experimental protocol, it does not establish clinical utility across different institutions, devices, or patient populations. Future studies should therefore investigate prospective clinical validation, real-world workflow integration, and deployment under heterogeneous imaging conditions. In parallel, the PMSL strategy could be further explored for other medical imaging modalities and resource-constrained deployment scenarios.

## 7 Conclusion

This study proposes a lightweight CXR multi-disease detection framework based on YOLOv8n, termed PMSL-YOLO. By integrating progressive multi-scale learning, a Freeze–Unfreeze staged training mechanism, and medical-image-oriented data augmentation, the proposed framework improves multi-disease detection performance while preserving the lightweight architecture of YOLOv8n. The framework also incorporates bounding-box visualization and Grad-CAM as supplementary post-hoc tools for qualitative model behavior and error analysis.

Evaluation on the completely untouched test set provides independent evidence within the present dataset and experimental protocol. PMSL-YOLO with the progressive 512→512→640→1024 configuration achieves 94.1% Precision, 92.4% Recall, 93.3% mAP@0.5, and 70.1% mAP@0.5:0.95, compared with 89.6%, 80.9%, 86.7%, and 68.4%, respectively, for the 1024 × 1024 YOLOv8n baseline. The results further indicate that PMSL improves detection of representative small-lesion categories, while the ablation and schedule-sensitivity analyses support the contribution of progressive resolution adaptation to the observed performance gains.

From a lightweight design perspective, PMSL-YOLO retains the original YOLOv8n architecture with only 3.2M parameters and introduces no additional inference-time network modules. The progressive training strategy therefore improves detection performance primarily through the training process rather than by increasing model capacity. Together with the measured inference efficiency, these characteristics indicate potential suitability for resource-constrained CXR detection applications. The Grad-CAM visualizations provide supplementary qualitative information for model behavior analysis, but should not be interpreted as clinically validated explanations.

Overall, PMSL-YOLO provides a lightweight training framework that achieves a favorable balance between detection performance and computational efficiency under the present experimental conditions. The independent test evaluation strengthens the evidence that the observed gains are not confined to the validation subset used during model development. However, the current evidence remains limited to a single public dataset and a single random seed, and does not establish robustness across institutions, imaging devices, patient populations, or real clinical workflows. Future work should therefore focus on external and multi-center validation, repeated-seed evaluation, prospective clinical assessment, and further investigation of PMSL across diverse medical imaging tasks. The ultimate goal is to develop reliable AI-assisted diagnostic tools that complement rather than replace clinical decision-making.

## Data Availability

Publicly available datasets were analyzed in this study. This data can be found here: The dataset used in this study is the publicly available VinBigData Chest X-ray Abnormalities Detection dataset. It can be accessed at: https://physionet.org/content/vindr-cxr/1.0.0/Repository: PhysioNet.
